# Subepithelial myofibroblasts enhance stem cell regeneration after intestinal epithelial damage

**DOI:** 10.1242/dev.205629

**Published:** 2026-09-29

**Authors:** Neil McCarthy, Camila Mendez Jimenez, Guodong Tie, Gizelle McCarthy, Shariq Madha-Krause, Ruiyang He, Ramesh A. Shivdasani

**Affiliations:** ^1^ Department of Molecular Biomedical Sciences, College of Veterinary Medicine, North Carolina State University, Raleigh, NC 27502, USA; ^2^ Department of Medical Oncology and Center for Functional Cancer Epigenetics, Dana-Farber Cancer Institute, Boston, MA 02215, USA; ^3^ Department of Medicine, Harvard Medical School, Boston, MA, 02215, USA; ^4^ Harvard Stem Cell Institute, Cambridge, MA 02139, USA

**Keywords:** Intestinal stem cell regeneration, Niche-stem cell interactions, Subepithelial myofibroblasts, PDGFRA fibroblasts, Telocytes

## Abstract

When intestinal stem cells (ISCs) are damaged, other epithelial cells de-differentiate to restore the ISC compartment. Immune, nerve and lymphatic endothelial cells contribute signals that promote this response, but it is unclear whether or how the native mesenchymal niche senses and responds to ISC loss. In homeostasis, trophocytes and smooth muscle beneath intestinal crypts secrete BMP inhibitors (BMPi) and R-spondins to foster ISC self-renewal, whereas sub-epithelial myofibroblasts (SEMFs) concentrated at crypt tops promote differentiation through bone morphogenetic proteins (BMPs). We assessed transcriptional responses in mouse sub-epithelial populations after ablating ISCs with whole-body irradiation and treating *Lgr5^DTR^* mice with diphtheria toxin. More than any other cell type, SEMFs modulated expression of genes related to ISC expansion. However, the BMPi gene *Smoc1* and non-canonical Wnt signals, both expressed in SEMFs after whole-body irradiation, were dispensable for ISC restoration. Instead, SEMFs appear to support ISC regeneration through factors that are not induced by ISC attrition per se but by local inflammation. These findings reveal unexpectedly limited transcriptional modulation of the native niche after ISC losses and context-dependent signaling functions for SEMFs.

## Teaser

**Summary:** Intestinal subepithelial myofibroblasts normally oppose epithelial stem cell properties. Following damage to intestinal stem cells, however, they provide inflammation-induced signals that promote stem cell regeneration.

## INTRODUCTION

Intestinal homeostasis depends on signaling gradients – including Wnts, Rspondins (RSPOs), and BMP agonists and inhibitors (BMPi) – established by a polarized mesenchyme (Clevers, 2026). Positioned at the base of epithelial crypts, PDGFRA^lo^ fibroblasts express CD81 (trophocytes) and/or CD55, and PDGFRA**^−^** muscularis mucosae (MM) express BMPi and RSPOs to support intestinal stem cell (ISC) and progenitor replication (McCarthy et al., 2020; Kraiczy et al., 2023). Conversely, PDGFRA^hi^ sub-epithelial myofibroblasts (SEMFs, also known as telocytes) concentrated at crypt and villus tops express BMPs, which promote epithelial differentiation (McCarthy et al., 2023). Following injury, ISCs regenerate through de-differentiation, a process facilitated by Wnts, RSPOs, BMPi and other mesenchymal and immune cell products (Hageman et al., 2020). Specific contributions of native niche cells to this restoration remain unknown.

We examined resident niche cell responses after whole-body radiation or selective ISC ablation in mice. Within the transcriptional response to radiation, trophocytes and muscularis mucosae were comparatively inert, while SEMFs modulated many growth factors. Among induced genes, *Smoc1* showed modest BMPi activity *in vitro* but was dispensable *in vivo*. Mice defective in Wnt delivery from SEMFs and smooth muscle tolerated radiation-induced ISC loss, and substantial SEMF depletion had little impact on ISC numbers or proliferation in homeostasis or regeneration, but SEMFs supported ISC regeneration in organoid co-cultures. Pro-inflammatory cytokines triggered expression of some regenerative SEMF products and augmented that effect. On a background of modest transcriptional modulation in the native niche, these findings reveal contextual SEMF functions after ISC injury.

## RESULTS AND DISCUSSION

### ISC regeneration *in vitro* depends on growth factors

Because ablation of BMPi/RSPO-expressing subepithelial cells in mice causes rapid ISC attrition and is lethal (McCarthy et al., 2020), we tested requirements for BMPi and different amounts of RSPO in ISC regeneration in crypt-derived organoids that received 4 Gy γ-irradiation (Fig. 1A). At the lowest concentration of RSPO1 that sustained unirradiated organoids (1% conditioned medium), budding – which signifies ISC activity (Sato et al., 2009) – was attenuated in irradiated organoids (Fig. 1A), indicating that some Wnt/RSPO signaling is necessary to restore ISCs. Removal of recombinant (r) noggin (NOG), which is a BMPi, extinguished budding and reduced cell replication – effects that were negated at high RSPO1 concentration (10% conditioned medium, Fig. 1A and Fig. S1A). Whether organoids were irradiated or not, BMPi induced ISC genes *Lgr5* and *Olfm4*, as expected, and had small effects in irradiated organoids on genes associated with ISC regeneration (Ayyaz et al., 2019), e.g. *Ly6a* and *Ly6d* (Fig. S1B). Thus, ISC restoration in organoids is sensitive to both RSPO and BMPi levels, likely reflecting requirements *in vivo*.

**Fig. 1.**
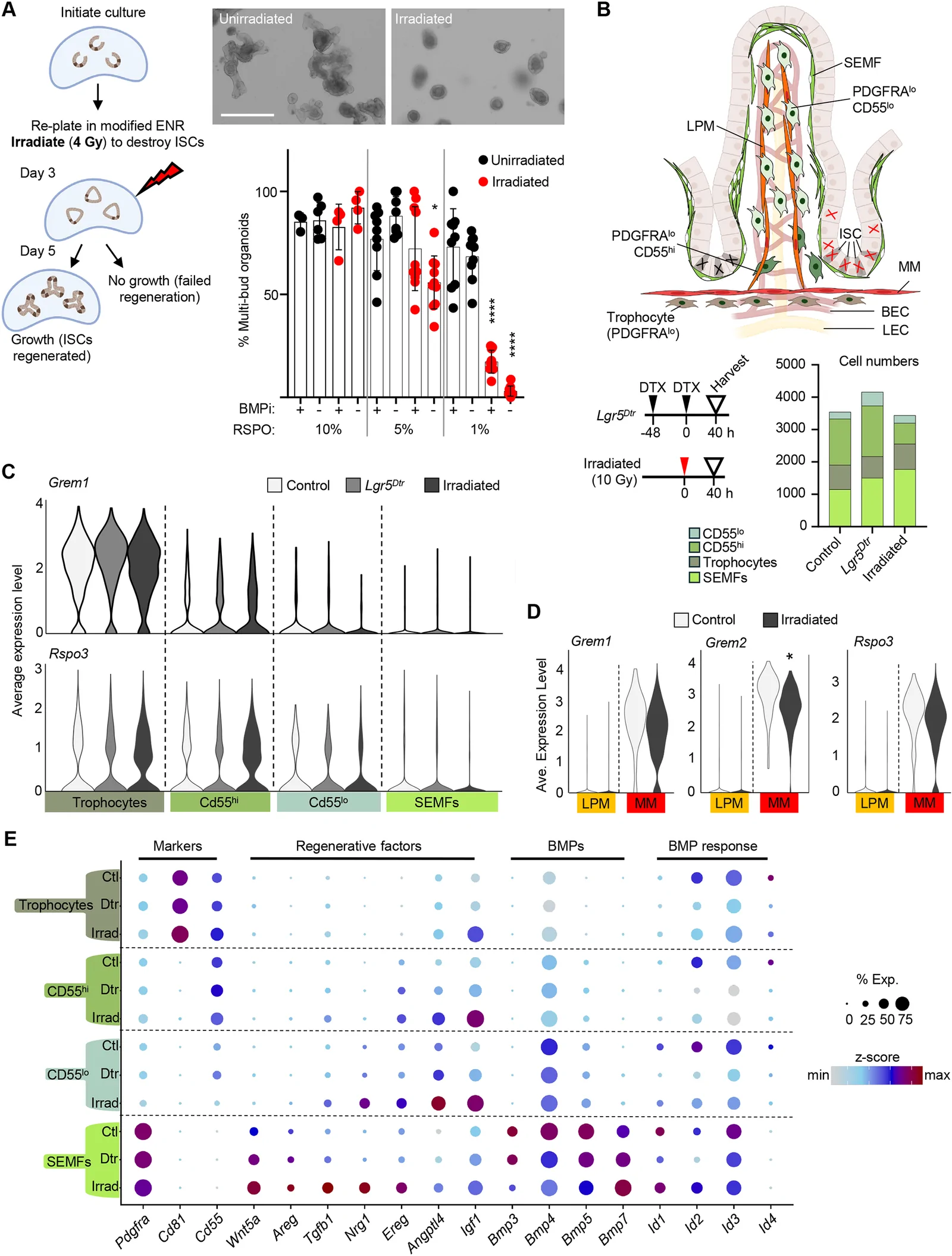
**Analysis of growth factor requirements in intestinal stem cell regeneration *in vitro* and gene expression in mesenchymal niche cells following intestinal stem cell loss.** (A) Experimental design for organoid regeneration. Representative organoids from 1% RSPO+rNOG (BMP inhibitors; BMPi) are shown. Scale bar: 50 μm. Graph shows the percentages of organoids with more than 2 buds (128±17 organoids in unirradiated controls). Data are mean±s.e.m. Significance was determined within each RSPO concentration group by one-way ANOVA followed by Tukey's multiple comparisons test and is reported relative to unirradiated (+BMPi) controls. ****P<0.0001, *P<0.05. (B) Two modes of intestinal stem cell (ISC) loss were used to examine niche responses: Lgr5^+^ cell ablation (black crosses) and γ-irradiation (red crosses). Sub-epithelial myofibroblasts (SEMFs) and PDGFRA^lo^ cells were isolated for bulk and single-cell RNA sequencing from *Pdgfra^H2BeGFP^* mice. After irradiation, blood (BEC) and lymphatic (LEC) endothelial cells were isolated for bulk RNA-seq and smooth muscle populations (lamina propria myocytes, LPM; muscularis mucosae, MM) for scRNA-seq. Graph shows the proportions of each PDGFRA^+^ population isolated under the indicated conditions. (C) Normalized expression (scRNA-seq) of *Grem1* and *Rspo3* across cell types and conditions. (D) *Grem1*, *Grem2* and *Rspo3* expression in smooth muscle populations after irradiation (Wilcoxon, *P<0.05). LPM, lamina propria myocytes; MM, muscularis mucosae. (E) Relative scRNA-seq values of cell markers and niche factors (stimulatory or repressive) across treatments from integrated *Pdgfra^H2BeGFP^* datasets. Circle size represents the percentage of cells expressing each gene; colors represent scaled average expression. (A,B) Schematics created in BioRender by McCarthy, N., 2026. https://BioRender.com/x74v102. This figure was sublicensed under CC-BY 4.0 terms.

### Transcriptional modulation of subepithelial cells after ISC loss

The native mesenchyme is presumed to sense ISC loss and send signals for ISC regeneration. To identify transcriptional responses to ISC loss, we generated bulk RNA sequencing libraries of distinct PDGFRA^+^ fibroblast populations isolated from *PDGFRA^H2BeGFP^* mice: trophocytes, SEMFs, PDGFRA^lo^CD55^hi^ and PDGFRA^lo^CD55^lo^ (Fig. 1B). We implemented two modes of ISC loss−10 Gy whole-body irradiation (Metcalfe et al., 2014) and injection of diphtheria toxin into *Lgr5^Dtr^* mice (Tian et al., 2011) – and harvested cells at the approximate onset of ISC restoration (Fig. 1B). As controls, we isolated bulk blood and lymphatic endothelial cells (CD31^+^LYVE^−^ BECs and CD31^+^LYVE1^+^ LECs, respectively) from irradiated mice, to compare with the same populations from unirradiated mice (GEO: GSE130681). RNA-seq data were concordant among duplicate samples across populations and treatments, and marker genes confirmed reliable FACS sorting (Fig. S1C). We also isolated GFP^−^ (PDGFRA^−^) tdTom^+^ *Myh11^+^* lamina propria myocytes (LPM) and muscularis mucosae (MM) from irradiated *Pdgfra^H2BeGFP^;Myh11^Cre(ER−T2)^;Rosa26^L-S-L-tdTom^* mice (Fig. 1B and Fig. S1D) (McCarthy et al., 2023).

Because ISC maintenance *in vivo* and regeneration *in vitro* (Fig. 1A) rely on Wnt/Rspo/BMPi signaling, we focused on these pathways. We observed little bulk change in *Rspo3*, *Wnt2* or *Wnt2b* in endothelial or PDGFRA^lo^ cells, and mixed responses among Wnt inhibitors *Frzb* (increased) and *Dkk2* (decreased) in bulk PDGFRA^lo^ cells (Fig. S1E,F and Table S1). Whole-body radiation has effects beyond ISC ablation, so some of these alterations may not reflect ISC loss per se; indeed, selective ISC ablation in *Lgr5^Dtr^* mice elicited modest mRNA changes in SEMFs or PDGFRA^lo^ cells (Fig. S1C). Because changes in select cells could be masked in bulk cell isolates, we examined over 3000 *Pdgfra^H2B-eGFP+^* cells from *Lgr5^Dtr^* and irradiated mice, then integrated the findings with control data (GEO: GSE130681); we recovered similar fractions of each PDGFRA^+^ cell type across treatments (Fig. 1B). Consistent with bulk RNA-seq findings, irradiation elicited more changes than *Lgr5^Dtr^* ablation (Fig. S1G and Table S1). *Grem1* and *Rspo3* were not materially affected in trophocytes or CD55^hi^ cells following ISC loss (Fig. 1C), no other BMPi or Wnt genes were induced, and *Dkk2* decreased in CD55^hi^ cells (Fig. S2A). MM from irradiated mice, which are *Myh11*^+^*Pitx1*^+^*Grem2*^+^ compared to LPM (Fig. S2B), showed reduced *Grem2* and no change in *Grem1* or *Rspo3* compared to untreated mice (Fig. 1D) (GEO: GSE130681 and GSE184158); however, non-canonical *Wnt4* was increased in LPM (Fig. S2C and Table S1).

By contrast, SEMF-expressed growth factor genes showed numerous changes, most notably after irradiation, including increased expression of non-canonical *Wnt5a* and regenerative factors *Areg*, *Tgfb1*, *Ereg* and *Angptl4* (Fig. 1E and Fig. S1F; Chen et al., 2023; Fink et al., 2024; Miyoshi et al., 2012; Shao and Shang, 2010). *Igf1* increased after irradiation in all fibroblast groups, and *Nrg1* increased in CD55^lo^ cells and SEMFs. Bulk and scRNA-seq showed reduced *Bmp3*, *Bmp4* and *Bmp5*, and increased *Bmp7* (Fig. 1E and Fig. S1F); following ISC loss, *Id* transcription factors, which are targets of BMP signaling (Miyazono et al., 2005), were reduced across PDGFRA^+^ cell types and smooth muscle (Fig. 1E and Fig. S2C). Thus, following ISC loss, factors essential for resting ISC function are transcriptionally unaltered in trophocytes or MM, whereas SEMFs – which oppose rather than support resting ISCs (McCarthy et al., 2023) – show the most modulation of genes pertinent to niche functions, including growth factors and, especially, BMP signaling components.

### BMPi and Wnt activity from SEMFs is dispensable during ISC regeneration

One gene increased in SEMFs in bulk and single-cell RNA-seq following both forms of ISC loss was *Smoc1*, and *in situ* hybridization confirmed elevated expression in SEMFs in irradiated mice (Fig. 2A). Because *Smoc1* is a putative atypical BMPi (Rainger et al., 2011), we examined this possibility in intestinal organoids. Although less potent than rNOG, rSMOC1 promoted organoid growth at high concentrations and, compared to organoids cultured without BMPi, induced ISC marker genes (Fig. 2B). *Smoc1* mutant mice have complex defects and mutations in humans are associated with microphthalmia and limb anomalies (Okada et al., 2011) but intestinal *Smoc1* functions are unknown. To evaluate possible roles in ISC restoration, we exposed *Pdgfra^Cre(ER-T2)^;Smoc1^Fl/Fl^;Rosa26^L-S-L-tDtom^* mice to 10 Gy whole-body radiation 2 weeks after giving the first dose of tamoxifen to delete *Smoc1* (Fig. 2C). Tamoxifen-injected animals showed *Smoc1* deletion in Cre-expressing tdTom^+^ cells and absence of *Smoc1* 40 h post-irradiation (hpi, Fig. S2D). We observed no overt regenerative deficits, and *Olfm4*^+^ cell numbers and crypt cell proliferation at 40 and 96 hpi, respectively, were the same as in control animals (Fig. 2C and Fig. S2E). Thus, *Smoc1* displays BMPi activity *in vitro*, but despite increased SEMF expression after ISC loss, is not required for crypt homeostasis or ISC regeneration *in vivo*; this may indicate that *Smoc1* is redundant in the presence of other niche factors*.*

**Fig. 2.**
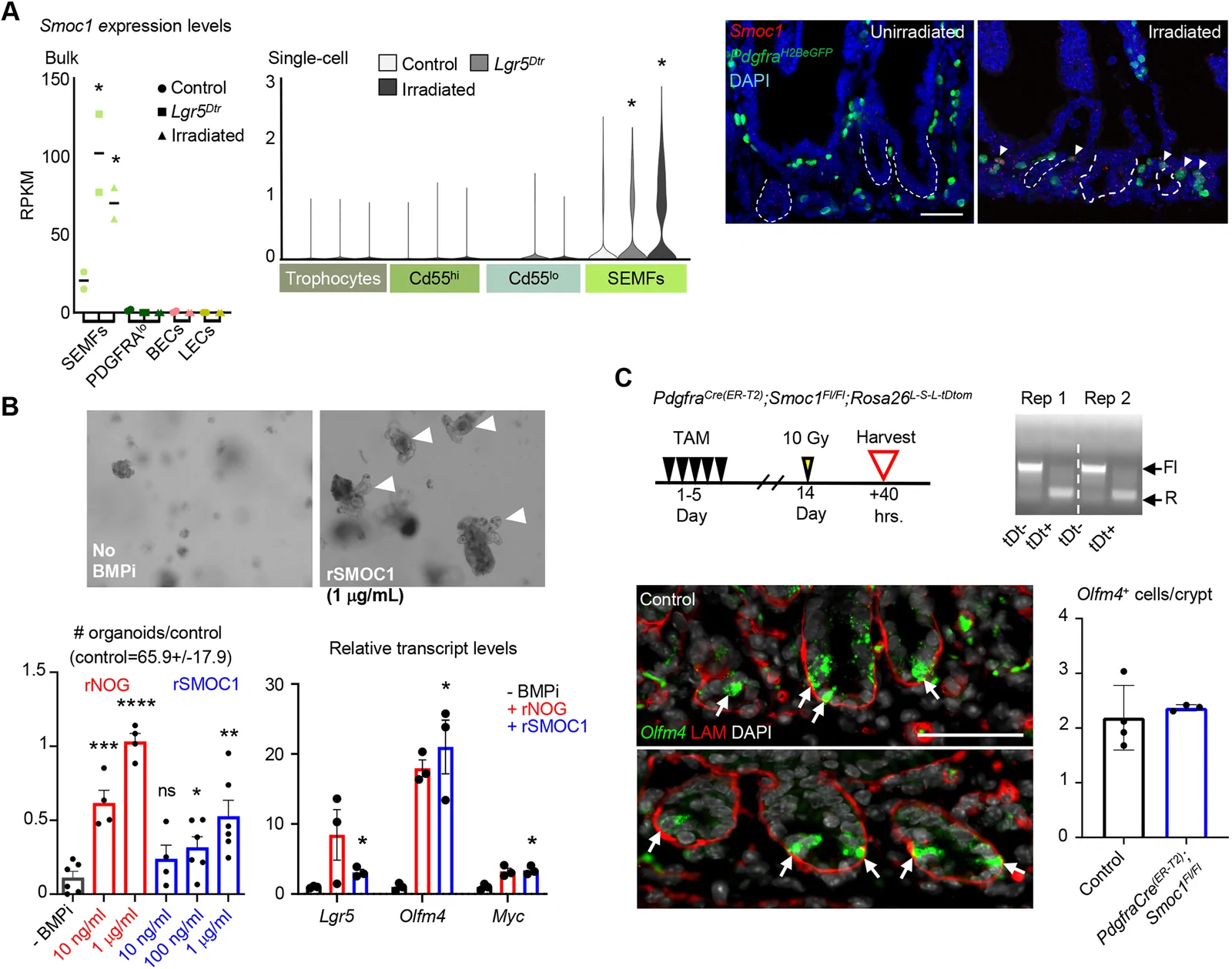
**SMOC1 acts as a redundant BMP inhibitor and is dispensable during intestinal stem cell regeneration.** (A) Bulk (left) and single-cell (right) RNA-seq data show increased *Smoc1* expression in sub-epithelial myofibroblasts (SEMFs) in both intestinal stem cell (ISC) loss conditions (Wald and Wilcoxon tests, respectively, *P<0.05). Representative images of *Smoc1* RNA *in situ* hybridization show SEMF localization (arrowheads) following irradiation. Dashes outline individual crypts. Scale bar: 50 μm. (B) Representative images of organoids cultured with recombinant (r) SMOC1 (right) or no BMP inhibitor (BMPi; left). qRT-PCR analysis of organoids in indicated conditions (*n*=4-6 biological replicates, control condition: 100 ng/ml rNOG; data are mean±s.e.m.). Transcript levels are represented relative to organoids without BMPi (*n*=3). Significance was determined using an unpaired, two-tailed *t*-test. *P<0.05, **P<0.01, ***P<0.001, ****P<0.0001. (C) *Pdgfra^Cre(ER-T2)^;Smoc1^Fl/Fl^;Rosa26^TdTom^* mice were injected with tamoxifen (TAM) for 5 consecutive days and irradiated 2 weeks after the first injection. Genomic PCR confirms efficient recombination of the floxed allele (lower band) in FACS-sorted TdTom^+^ compared to TdTom^−^ cells. Bottom: *Olfm4 in situ* hybridization image of *Pdgfra^Cre(ER-T2)^;Smoc1^Fl/Fl^* small intestine 40 h after irradiation. Arrows indicate Olfm4^+^ cells. Graph shows the number of *Olfm4*^+^ cells/crypt (*n*=3 or 4, ≥25 crypts/sample; data are mean±s.e.m.). An unpaired, two-tailed *t*-test revealed no significance. Scale bar: 50 μm.

SEMFs are a proposed source of canonical Wnts (Shoshkes-Carmel et al., 2018) but deletion of *Porcn*, a gene required for Wnt secretion (Proffitt and Virshup, 2012), in SEMFs does not elicit epithelial defects at rest; rather, PDGFRA^lo^ fibroblasts are principal sources of Wnts, RSPOs and BMPi (Kraiczy et al., 2023; San Roman et al., 2014). Canonical Wnts and non-canonical *Wnt5a* are, however, reportedly necessary for epithelial repair after acute colitis or mechanical wounding (Kabiri et al., 2014; Das et al., 2022; Saha et al., 2016; Miyoshi et al., 2012). After ISC losses, *Wnt4* and *Wnt5a* were elevated in LPM and SEMFs, respectively (Fig. S2C and Fig. 1E), while *Dkk2*, a Wnt inhibitor (Li et al., 2002), was reduced in PDGFRA^lo^Cd55^hi^ fibroblasts (Fig. S2A). These findings suggested that Wnt signaling from SEMFs and/or smooth muscle may be dispensable for resting ISC function but might help restore ISCs after injury. To delete *Porcn* in SEMFs and smooth muscle, we generated *Pdgfra^Cre(ER-T2)^;Myh11^Cre(ER-T2)^;Porcn^Fl/Fl^* conditional mutant (*Porcn^PMKO^*) mice. A *Rosa26^L-S-L-tDtom^* allele reported Cre recombinase activity and PCR analysis of DNA from *Porcn^PMKO^*;*Rosa26^L-S-L-tDtom+^* cells confirmed efficient recombination (Fig. S3A). mRNA from *Porcn^PMKO^* crypt cells showed insignificant changes in ISC marker genes (Fig. S3B) and 96 h after γ-irradiation, ISCs were efficiently restored in mutant mice (Fig. S3C). *Porcn in situ* hybridization revealed little mesenchyme signal compared to the epithelium in both control and *Porcn^PMKO^* mice (Fig. S3D), suggesting that epithelial Wnts might compensate for mesenchymal absence of *Porcn* function.

### SEMF ablation and *in vitro* characterization

The seeming lack of an SEMF requirement for ISC restoration is unsurprising because SEMFs oppose the ISC state *in vitro* (McCarthy et al., 2023). However, substantial mRNA alterations after ISC attrition included *Tgfb1* and other regenerative factors: *Areg*, *Angptl4*, *Nrg1*, *Ereg* and *Igf1* (Fig. 1E). To test whether SEMFs serve as a contextual source of regenerative signals, we ablated SEMFs with diphtheria toxin in *Pdgfra^Cre(ER-T2)^;Rosa26^mTmG/iDtr^* mice and irradiated mice 2 days after the last of three toxin injections, which had reduced GFP^+^ fibroblast numbers (Fig. 3A). ISC markers *Lgr5* and *Olfm4* and crypt cell proliferation were unaffected, and *Olfm4*^+^ crypts or cell proliferation showed little change 72 h after irradiation (Fig. 3B and Fig. S3E,F). Among markers of ISC regeneration, *Ly6a*, but not *Tgfbr2*, decreased slightly (Fig. S4A,B). This lack of epithelial defects warranted accurate estimation of PDGFRA^+^ cell reduction, but *Pdgfra^H2BeGFP^* and *Pdgfra^Cre(ER-T2)^* are knock-in alleles, so compound mice are *Pdgfra* null and non-viable. We therefore compared cells labeled in *Pdgfra^Cre(ER-T2)^* versus *Pdgfra^H2BeGFP^* mice by identifying corresponding anatomical regions and SEMFs using ACTA2 (encoding α-smooth muscle actin) and PDGFRA immunohistochemistry, respectively (Fig. 3C and Fig. S4C,D).

**Fig. 3.**
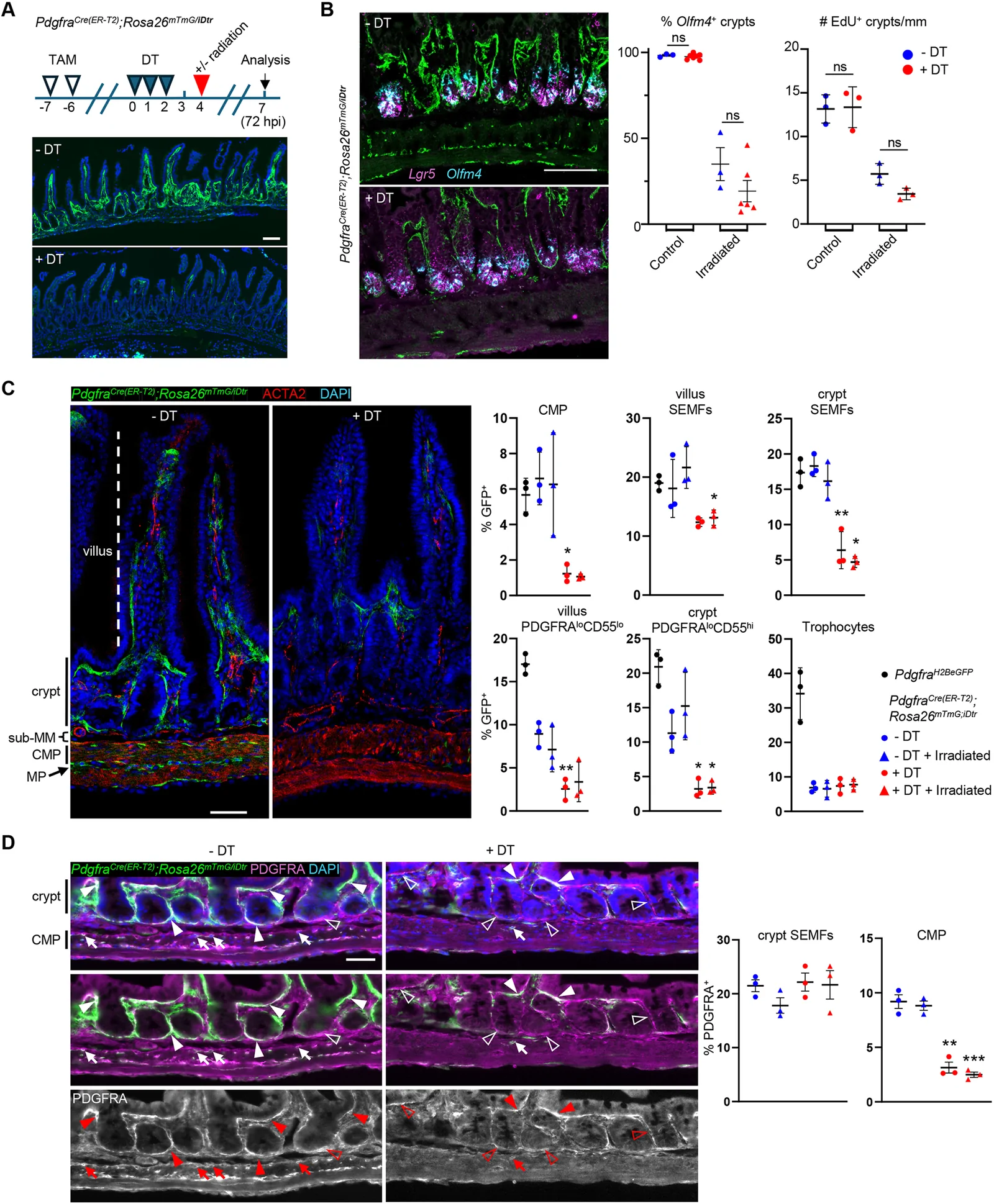
**Crypt homeostasis and regeneration following sub-epithelial myofibroblast ablation.** (A) *Pdgfra^Cre(ER-T2)^;Rosa26^mTmG/iDtr^* mice were injected with tamoxifen (TAM) on 2 consecutive days and, 1 week later, with diphtheria toxin (DT) for 3 consecutive days. Mice were irradiated 48 h after the final DT dose and analyzed at 72 h thereafter. Images show GFP^+^ cell depletion in DT-injected or uninjected mouse ilea. Scale bar: 100 μm. (B) Left: *in situ* hybridization of intestinal stem cell loss (ISC) markers *Lgr5* and *Olfm4* in ileal tissue sections from −DT and DT-injected *Pdgfra^Cre(ER-T2)^;Rosa26^mTmG/iDtr^* mice. Scale bar: 50 μm. Right: percentage of *Olfm4*^+^ crypts (>100 crypts/sample) and EdU^+^ crypts (>4 mm total length/sample) in indicated conditions, including 72 h post-irradiation (*n*=3-6; data are mean±s.e.m.). (C) Left: representative images of *Pdgfra^Cre(ER-T2)^;Rosa26^mTmG/iDtr^* mouse ileum stained for ACTA2 (encoding α-smooth muscle actin) after DT exposure (right) or no DT treatment (left). GFP depletion was quantified in the myenteric plexus (MP), circular muscularis propria (CMP), sub-MM (muscularis mucosae), and crypt and villus lamina propria. Scale bar: 50 μm. Right: quantitation of GFP^+^ nuclei or cells in the compartments defined in B (>4 mm total length/sample, 20 villi/sample, *n*=3/group; data are mean±s.e.m.). (D) Left: representative images of *Pdgfra^Cre(ER-T2)^;Rosa26^mTmG/iDtr^* mice stained for PDGFRA (filled arrowheads indicate crypt GFP^+^ PDGFRA^+^; open arrowheads indicate crypt GFP^-^PDGFRA^+^ cells; arrows indicate CMP PDGFRA^+^ cells). Scale bar: 50 μm. Right: quantitation of PDGFRA^+^ cells in crypt and CMP regions (>4 mm total length/sample, *n*=3; data are mean±s.e.m.). Significance was determined using an unpaired, two-tailed *t*-test between −DT and +DT conditions. *P<0.05, **P<0.01, ***P<0.001.

Compared with *Pdgfra^H2BeGFP^* mice, tamoxifen-treated *Pdgfra^Cre(ER-T2)^;Rosa26^mTmG/iDtr^* mice showed comparable numbers of *Pdgfra*-marked (GFP^+^) cells in the circular muscularis propria (CMP), myenteric plexus and SEMFs in both villus and crypt compartments; DT exposure eliminated cells in the CMP and reduced SEMFs <50% (Fig. 3C and Fig. S4D,E). PDGFRA^lo^ cells, including villus CD55^lo^ and crypt CD55^hi^, were inefficiently labeled in *Pdgfra^Cre(ER-T2)^* mice but reduced after DT treatment (Fig. 3C), while trophocytes beneath the muscularis mucosae (MM) were neither efficiently labeled nor reduced (Fig. 3C). Irradiation did not affect labeled cells in any PDGFRA^+^ compartment and scRNA sequencing of labeled cells revealed only PDGFRA^+^ fibroblasts (SEMFs, trophocytes, CD55^hi^ and CD55^lo^), excluding the possibility that *Pdgfra^Cre(ER-T2)^* mice inadvertently mark PDGFRA^-^ cells (Fig. 3C and Fig. S4F). Thus, although all PDGFRA^+^ cells other than trophocytes decreased after DT treatment, residual cells could account for intact ISC regeneration (Fig. 3C and Fig. S4D). Moreover, PDGFRA immunostaining showed that non-Cre labeled areas in DT-treated *Pdgfra^Cre(ER-T2)^;Rosa26^mTmG/iDtr^* mice were PDGFRA^+^, even while PDGFRA^+^ cells found in the external muscle were absent (Fig. 3D). Previous reports using *Col6a1*-Cre mice to ablate mesenchymal cells showed regeneration following cellular losses (Melissari et al., 2021), suggesting that partial recovery may also impact the lack of overt post-radiation defects in these experiments.

To circumvent these technical limitations, we used organoid co-cultures to assess SEMF activity in ISC regeneration. SEMFs produce BMPs, and organoids co-cultured with SEMFs lose ISCs and differentiate precociously (McCarthy et al., 2023), but SEMFs also express regenerative factors like TGFβ, which promote ISC regeneration (Chen et al., 2023 and Fink et al., 2024). In organoid cultures, TGFβ, BMP2 and SEMFs all suppressed ISC markers *Lgr5* and *Olfm4*, and, surprisingly, increased *Id1*, a marker of BMP activity, but differed in their effects on *Sox9* (Fig. 4A). SEMF co-cultures mimicked TGFβ, and not BMPs, in inducing regenerative markers *Anxa1*, *Ly6d* and *Tgfbr2*, albeit less potently than TGFβ did (Fig. 4A). Thus, SEMFs indeed elicit a TGFβ-like regenerative response in overlying epithelial cells, prompting us to ask whether stimuli associated with irradiation might enhance regenerative SEMF effects.

**Fig. 4.**
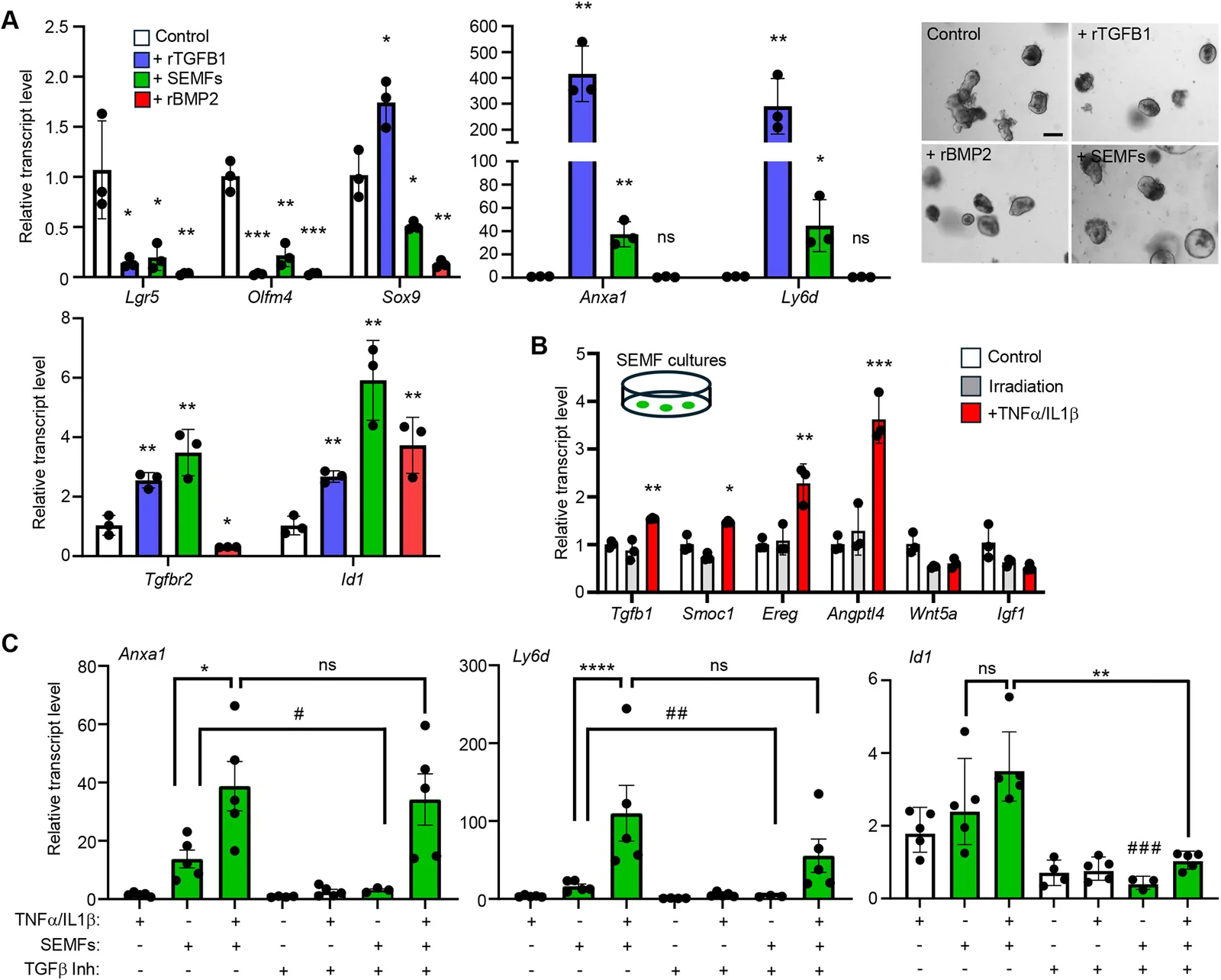
**Regenerative phenotype in organoids cultured with sub-epithelial myofibroblasts exposed to proinflammatory cytokines.** (A) qRT-PCR analysis of isolated organoids after indicated treatments (data are mean±s.e.m., *n*=3). Significance was determined using an unpaired, two-tailed *t*-test relative to controls; ***P<0.001, **P<0.01, *P<0.05. Images show representative organoids grown for 24 h in ENR medium with rBMP2, rTGFB1 or sub-epithelial myofibroblasts (SEMFs). Scale bar: 50 μm. (B) qRT-PCR analysis of regenerative factor mRNAs expressed in SEMFs 24 h after irradiation or treatment with IL1β and TNFα (mean±s.e.m., *n*=3). Significance was determined using unpaired, two-tailed *t*-test relative to untreated controls; ***P<0.001, **P<0.01, *P<0.05. (C) qRT-PCR analysis of organoids isolated after the indicated treatments (mean±s.e.m.). Significance was determined by one-way ANOVA followed by Tukey's multiple comparisons test. ****P<0.0001,**P<0.01, *P<0.05 (+SEMFs +TNFα/Il1β versus +SEMFs); ^###^*P*<0001, ^##^*P*<0.01, ^#^*P*<0.05 (+SEMFs +TGFβ inhibitor versus +SEMFs). *n*=3-5 independent organoid cultures.

Irradiation elicits an inflammatory response (Moraitis et al., 2025) that includes the cytokines IL1β and TNFα (Linard et al., 2003). SEMFs exposed to these pro-inflammatory cytokines, but not γ-irradiated SEMFs, induced *Tgfb1*, *Ereg* and *Angptl4* mRNAs (Fig. 4B). To test if IL1β and TNFα influence SEMF activity in restoring ISCs, we cultured organoids with SEMFs or with the cytokine cocktail, or with both. The cytokines alone did not induce regenerative genes. Instead, they substantially augmented SEMF-induced activation of *Anxa1* and especially *Ly6d* (activation of *Ly6a* was not statistically significant) without affecting ISC markers *Olfm4*, *Lgr5* or *Sox9* (Fig. 4C and Fig. S5A). The TGFβ inhibitor LY2109761 (Melisi et al., 2008) blocked the regenerative RNA response (and reduced *Id1*, but not *Tgfbr2* levels) in TGFβ-treated organoids (Fig. S5B). Notably, while LY2109761 abrogated the effects of cytokine-naïve SEMFs, it did not affect induction of regenerative genes by cytokine-exposed SEMFs (Fig. 4C). Thus, different stimuli induce different genes in the ISC regenerative response; moreover, the regenerative potential of cytokine-naïve SEMFs is TGFβ dependent but substantial cytokine augmentation of that activity is not.

### Conclusions

ISC homeostasis relies on distinct cell types to maintain requisite signal gradients. We asked whether acute ISC loss alters cells that maintain those physiologic gradients. SEMFs increased RNAs encoding several secreted growth factors previously identified as ‘regenerative’. Pro-inflammatory cytokines enhance the regenerative response in organoids co-cultured with SEMFs, suggesting that inflammation likely contributes to SEMF-mediated ISC rescue *in vivo*. Indeed, analysis of markers of inflammatory fibroblasts found in the colon, including *Mmp10*, *Il11*, *Ccl2*, and *Col18a1* (Cadinu et al., 2024), were also increased in our irradiated SEMF RNA-seq datasets (Table S1). The native niche cell response to ISC loss from γ-radiation or other damage is multifaceted and mediated in part by TGFβ-independent effects of pro-inflammatory cytokines on SEMFs. Although *in vitro* studies do not fully recapitulate the native SEMF response or the *in vivo* environment after epithelial damage, they allowed us to examine defined SEMF-expressed factors and revealed that factors in addition to TGFβ constitute the regenerative niche response. TGFβ expression is not exclusive to SEMFs (Chen et al., 2023), which also showed transcriptional shifts in BMP pathway components, including induction of the BMPi *Smoc1*. Given that BMP and TGFβ signaling often display reciprocal inhibition (Fink and Wrana, 2023 and Wu et al., 2024), altered BMP signaling may indirectly amplify TGFβ signaling to restore ISCs, as previously described in regenerating hair follicle stem cells (Oshimori and Fuchs, 2012).

## MATERIALS AND METHODS

### Animals

*Pdgfra^H2B-eGFP^* (JAX strain 00766), *Pdgfra^Cre(ER-T2)^* (JAX 032770), *Myh11^Cre(ER-T2)^* (JAX 019079), *Rosa26^RLSL-tdTomato^* (JAX 007909), *Rosa26^RLSL-mTmG^* (JAX 007676) and *Rosa26^RLSL-iDtr^* (JAX 007900) mouse lines were purchased from Jackson Laboratories. *Lgr5^Dtr-eGFP^* (Tian et al., 2011) and *Prcn^Fl/y^* (Barrott et al., 2011) have been described previously. *Smoc1^tm1c(EUCOMM)Wtsi>/H^* mice (referred to as *Smoc1^Fl/Fl^*) animals were purchased from the European Mouse Mutant Archive (EMMA, strain #04902). Adult mice were 8-10 weeks of age at the time of treatments or cell isolations. Animals were housed in a specific pathogen-free barrier facility, maintained on a 12 h light/dark cycle, and had *ad libitum* access to standard chow and water. All experiments used mice of both sexes and littermate controls. Animal procedures and experiments were approved and monitored by the Animal Care and Use Committee at the Dana-Farber Cancer Institute or North Caroline State University College of Veterinary Medicine.

### Mouse treatments

Adult mice received 1 dose of tamoxifen (Sigma, T5648, 1 mg per 25 g body weight) by intraperitoneal (IP) injection on 5 (*Pdgfra^Cre(ER-T2)^;Smoc1^Fl/Fl^*) or 2 consecutive days to induce recombination at *LoxP* sites and were harvested at times indicated in figure legends. BrdU (Sigma, B5002) and EdU (Thermo, C10340) was administered by IP injection 1 h before euthanasia (10 μg/g body weight). To ablate ISCs in *Lgr5^Dtr-eGFP^* mice, we injected diphtheria toxin (Sigma, D0564) IP at 50 μg/kg for two doses on alternate days and harvested animals 40 h after the second dose. To ablate SEMFs, tamoxifen-injected *Pdgfra^Cre(ER-T2)^;Rosa26^mTmG/iDtr^* mice received IP injections of diphtheria toxin (50 μg/kg) on 3 consecutive days. 10 Gy whole-body irradiation was delivered from a ^137^Cs source.

### Immunohistochemistry

Immunohistochemistry was performed on tissues fixed in 4% paraformaldehyde and embedded in OCT compound (Tissue-Tek, 4583). 10 μm sections were prepared using a Leica CM3050 cryostat. The following antibodies (1:1000 dilution unless indicated) were used: anti-Laminin (Sigma, L9393), anti-GFP (Abcam, ab6662), anti-bromodeoxyuridine (BrdU Life Technologies, B23151), anti-α-smooth muscle actin (ACTA2; Abcam, ab5694), anti-PDGFRA (R&D Systems, AF1062), 1:100), and Alexa Fluor-conjugated goat anti-rat, goat anti-rabbit or donkey anti-goat IgG (Invitrogen, A11081, A21071 or A11058). EdU was stained according to the manufacturer's protocol (ThermoFisher, C10340). Whole mounts of *Pdgfra^Cre(ER-T2)^;Rosa26^RLSL-tdTomato^* tissues (Fig. S2A) were imaged as previously described (McCarthy et al., 2020; Bernier-Latmani and Petrova, 2016). All images were taken using a Leica SP5X laser scanning confocal microscope, Leica Thunder Imager or an Olympus VS200 microscope and processed using ImageJ Fiji (Schindelin et al., 2012) or Qupath (Bankhead et al., 2017) software.

### *In situ* hybridization

The RNAscope (Advanced Cell Diagnostics. ACD) method (Wang et al., 2012a) was used to perform mRNA *in situ* hybridization on adult intestines collected from at least three mice per treatment. Probes designed by ACD for *Lgr5* (312171), *Ly6a* (427571), *Olfm4* (311831), *Porcn* (404971), *Smoc1* (571511) and *Tgfbr2* (406241) were hybridized and samples were processed according to the RNAscope protocol. After hybridization, tissue sections from *Pdgfra^H2BeGFP^* or *Pdgfra^CreERT2^;Rosa26^mTmG^* samples were further processed for staining using anti-laminin (Sigma, L9393,1:1000) or anti-GFP (Abcam, ab6662, 1:100) antibodies, as previously described (McCarthy et al., 2023). Schematic illustrations in Fig. 1 were generated with BioRender.

### Organoid cultures

Crypts isolated from wild-type mice were plated for 2-3 days in complete basal media containing primocin, penicillin, streptomycin, N2, B27 and N-acetylcysteine, with EGF (Thermo Fisher, 50 ng/ml), RSPO (10% culture supernatant from 293T-HA-RSPO1-Fc cells) and noggin (Peprotech, 100 ng/ml), as previously described (Sato et al., 2009). Organoids were then removed from Matrigel using Cell Recovery Solution (Corning, CLS354253) for 10 min at 4°C, recovered by centrifugation at 150 ***g*** for 10 min, and 50-100 organoids were replated in 20 μl Matrigel droplets in 24-well tissue culture plates. The replacement medium contained all the above components, with specific modifications as indicated in figure legends and text: reduced RSPO1 (1, 5 or 10% culture supernatant from 293T-HA-RSPO1_Fc cells), addition or removal of 10-1000 μg/ml rNOG, or replacement of rNOG with rSMOC1 (R&D#5550, 10-1000 μg/ml). Organoids were irradiated at 4 Gy (^137^Cs source) immediately after addition of new components and analyzed 2 days later (Fig. 1A and Fig. S1A,B). Organoids described in Fig. 4A,C were maintained in basal culture medium (ENR) with addition of 2×10^4^ SEMFs (isolated as described below), rTGFB1 (R&D, #7666, 50 ng/ml), rBMP2 (Peprotech, #120-02, 50 ng/ml) or Ly2109761 (TGFβ inhibitor, R&D, #7864, 10 μM). Multi-budding and total organoids (Figs 1A, 2B and Fig. S5B) were counted in 3-5 experiments with 2 or 3 technical replicates per group per experiment. RNA was harvested from organoids for qRT-PCR analysis on 3-5 replicates per group; mRNA expression values are represented relative to *Gapdh* (2^-ΔCT^) and to organoids cultured without added factors or cells (complete basal media, EGF, 10% RSPO and 100 ng/ml noggin). To quantify EdU uptake (Fig. S1A), organoids were treated with 10 μM EdU solution and harvested 1 h later, followed by EdU immunohistochemistry according to the manufacturer's protocol (Thermo, C10340). Bright-field organoid images were captured using an Olympus CKX53 microscope.

### Mesenchymal cell isolation

Mesenchymal cells were isolated as previously described (McCarthy et al., 2020). Briefly, after manual stripping of external muscles and serosa, epithelium was denuded by shaking the tissue for 20 min at 37°C in pre-warmed Hank's balanced salt solution (HBSS, Life Technologies) containing 10 mM EDTA. The remaining tissue was rinsed with HBSS, minced using a scalpel and digested for 30 min at 37°C in 3 mg/ml collagenase II (Worthington, LS004176) diluted in HBSS containing 5% fetal bovine serum (FBS). Extracted cells were centrifuged at 300 ***g*** for 5 min and washed with FACS buffer (PBS containing 0.1%BSA), and leukocytes were depleted in ACK Lysis buffer (Gibco) for 3 min. Washed cells were resuspended in FACS buffer for Ab staining. To isolate blood and lymphatic endothelial cells, LYVE1 Ab (1:100, AngioBio 11-034) was applied for 30 min and cells were washed in FACS buffer, followed by further staining with FITC-conjugated anti-rabbit IgG (1:1000, Jackson ImmunoResearch 2337972) and APC-conjugated CD31 Ab (1:100, BD Biosciences 551262). Cells were detected and sorted on a Sony SH800z flow cytometer with DAPI gating to identify live cells.

### *In vitro* cultures

Unfractionated mesenchyme extracted from *Pdgfra^H2BeGFP^* mice was plated as previously described (McCarthy et al., 2020) in Dulbecco's modified Eagle medium (DMEM) and F12 medium (GIBCO) supplemented with 10% FBS, penicillin, streptomycin, Glutamax and HEPES buffer. Medium was replaced 24 h after plating. Cells were removed 5 to 7 days later using 1×TrypLE (Gibco, 5 min at 37°C), washed in FACS buffer and isolated by flow cytometry. SEMFs (GFP^hi^ fraction from *Pdgfra^H2BeGFP^* mice) were replated in the above medium. When SEMFs became confluent 3-5 days later, they were exposed to 4 Gy irradiation (^137^Cs source) or a cocktail of Il1β (R&D, 401-ML) and TNFa (R&D, 410-MT), each at 50 ng/ml for 24 h. qRT-PCR was performed on three replicates per group; mRNA values are represented relative to *Gapdh* (2^-ΔCT^) and to cells cultured without added factors or irradiation. All primer sequences used in this study for qRT-PCR analysis are listed in Fig. S5C.

### RNA-sequencing and analysis

RNA was extracted from cells isolated as described above, using Trizol Reagent (ThermoFisher), and purified using the RNeasy Microkit (QIAGEN). Total RNA (5-10 ng) was used to generate libraries with the SMART-Seq v4 Ultra Low Input RNA kit (Clontech). Libraries were sequenced on a NextSeq500 instrument (Illumina) to obtain 75 bp single-end reads. Data were analyzed using the VIPER pipeline with default settings (Cornwell et al., 2018) and aligned to mouse reference genome mm10 using the STAR aligner (Dobin et al., 2013). RPKM (reads per kilobase per million) values were generated from STAR counts, data quality was verified using RSeQC (Wang et al., 2012b). Statistical analyses were conducted on the R platform (https://www.R-project.org/). Data were normalized and differential gene expression (Wald test, *P_adj_* <0.05, log_2_ |fold-change| >1.5) was determined in the DESeq2 package (Love et al., 2014). Relationships among samples were assessed by principal component analysis (PCA) of DESeq2 rlog-transformed counts and the expression heatmap (Fig. S1F) was generated using Morpheus (Broad Institute).

### Single-cell RNA transcriptomics and analysis

To generate single-cell RNA libraries, we pooled 5000 to 10,000 cells at 1:2 ratio of PDGFRA^hi^ to PDGFRA^lo^ fibroblasts isolated by flow cytometry from *Pdgfra^H2BeGFP^* mice. After cells were loaded onto a Chromium Controller (10X Genomics), libraries were prepared according to the manufacturer's recommendations (Single Cell 3′ V3 assay) and sequenced on a HiSeq4000 instrument (Illumina). Libraries were aligned to the mm10 mouse transcriptome, unique molecular identifiers (UMIs) were counted using Cell Ranger (10X Genomics) and data were analyzed using the Seurat package in R (Butler et al., 2018). Cells with >1400 and <15,000 total transcripts, >900 and <4000 detected genes, and <10% mitochondrial transcripts were retained. Datasets were integrated using anchor-based integration workflows (Butler et al., 2018) and cell types were annotated based on previously established marker expression profiles (Kraiczy et al., 2023). The FindAllMarkers function in Seurat was used to determine differential gene expression using the Wilcoxon rank-sum test with parameters P_adj_ <0.05 and logfc.threshold>1 across cell types in treated and control conditions (Butler et al., 2018). Gene expression were generated using the “VlnPlot”, and “FeaturePlot” functions in Seurat. Dotplots depicting gene-wise scaled average expression were generated using code based on the “DotPlot” function within the ggplot2 package in R.

Smooth muscle cells were isolated as previously described (McCarthy et al., 2023) from tamoxifen-injected and irradiated *Myh11^CreERT2^;Pdgfra^H2BeGFP^;Rosa26^tDtom^* mice. FACS-sorted smooth muscle cells (GFP^−^ tDtom^+^) were processed and retained as described above. The resulting library was integrated with control data from untreated smooth muscle (GSE130681, GSE184158) and normalized. Expression of known cell markers (McCarthy et al., 2023) distinguished lamina propria myocytes from muscularis mucosae, and we separated these populations for differential RNA expression analysis using the “FindAllMarkers” function in Seurat with parameters P_adj_ <0.05 and logfc.threshold 0.25. To compare expression of known cell markers between PDGFRA fibroblasts and smooth muscle (Fig. S2B), we integrated scRNA libraries described above and used the “DotPlot” function within the ggplot2 package in R.

### Quantitation and statistical analysis

All statistical analyses were performed in Prism software package v10.6.1. The number of independent replicates and statistical methods are stated in figure legends. No sample size estimations or blinding were performed. To quantify GFP^+^ versus GFP^−^ nuclei or cells (Fig. 3C and Fig. S4E) in mesenchymal compartments of *Pdgfra^H2BeGFP^* and *Pdgfra^Cre(ER-T2)^;Rosa26^mTmG/iDtr^* samples, respectively, cells found in the CMP, sub-muscularis mucosae (MM) and crypts (between the bottom villus and the muscularis mucosae) were counted along a >4 mm length and reported as % total GFP^+^. PDGFRA immunohistochemistry was performed to identify SEMFs (GFP^hi^ in *Pdgfra^H2BeGFP^* mice) in both crypt and villus compartments. GFP^+^ nuclei/cells found in the myenteric plexus were reported as total #GFP^+^/mm length (>4 mm length). Villus was defined as the mesenchymal region from the top of the crypt to the top of the villus, and data reported as % GFP^+^ cells/nuclei (*n*=20 villus/sample).

## Supplementary Material



10.1242/develop.205629_sup1 Supplementary information

Table S1. Differentially expressed genes (DEG) in distinct mesenchymal cell types following intestinal stem cell loss.
