# Supplementary material for: Subepithelial myofibroblasts enhance stem cell regeneration after intestinal epithelial damage
Source: Development. 2026 Sep 29;153(18):dev205629. doi: 10.1242/dev.205629 (PMC13677008; doi:10.1242/dev.205629)
Supplement: Supplementary file 1 [file develop-153-205629-s1.pdf]

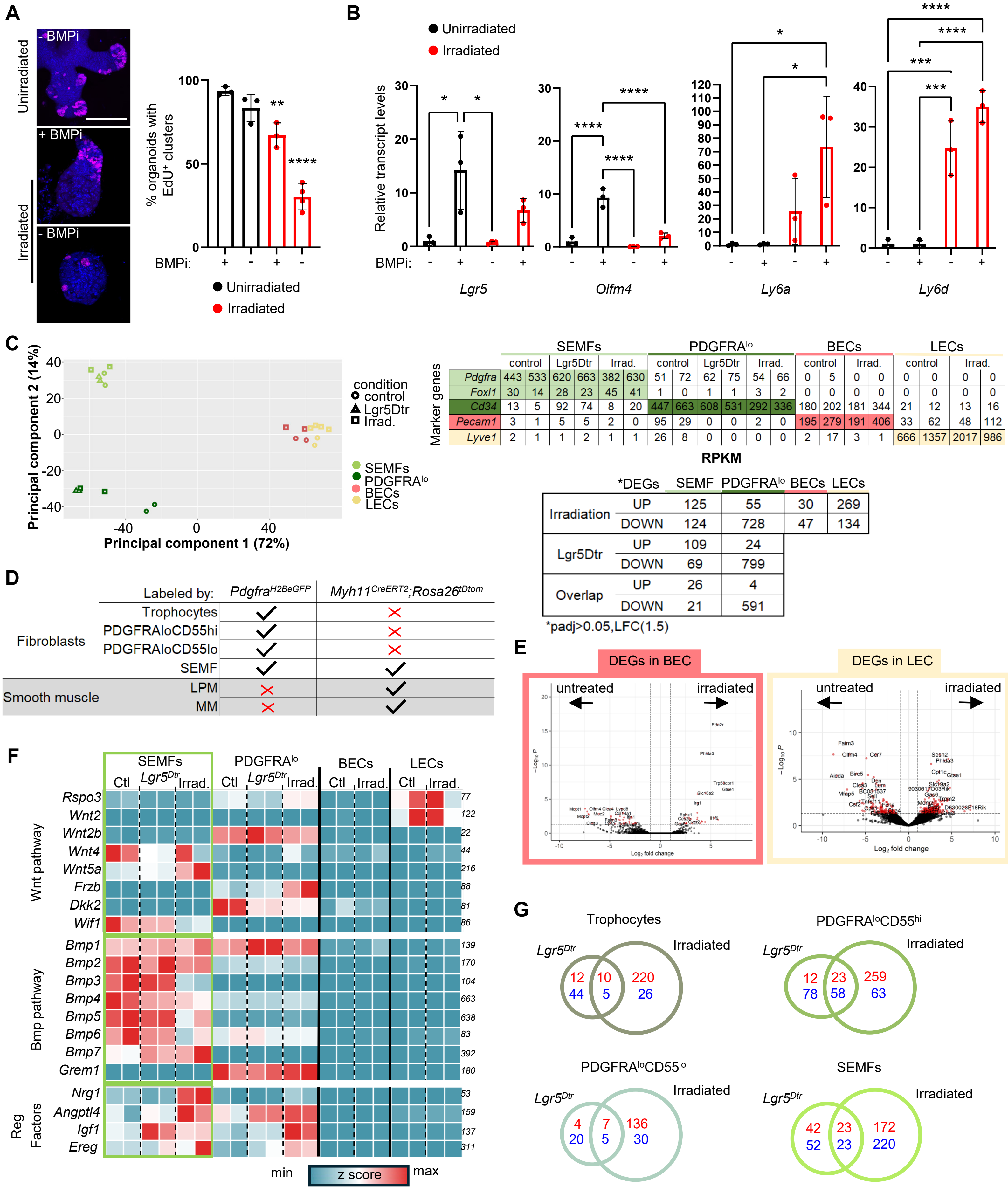

	SEMFs						PDGFRAlo						BECs				LECs			
	control		Lgr5Dtr		Irrad.		control		Lgr5Dtr		Irrad.		control		Irrad.		control		Irrad.	
Pdgfra	443	533	620	663	382	630	51	72	62	75	54	66	0	5	0	0	0	0	0	0
Foxl1	30	14	28	23	45	41	1	0	1	1	3	2	0	0	0	0	0	0	0	0
Cd34	13	5	92	74	8	20	447	663	608	531	292	336	180	202	181	344	21	12	13	16
Pecam1	3	1	5	5	2	0	95	29	0	0	2	0	195	279	191	406	33	62	48	112
Lyve1	2	1	1	2	1	1	26	8	0	0	0	0	2	17	3	1	666	1357	2017	986

RPKM

*DEGs		SEMF	PDGFRAlo	BECs	LECs
Irradiation	UP	125	55	30	269
	DOWN	124	728	47	134
Lgr5Dtr	UP	109	24		
	DOWN	69	799		
Overlap	UP	26	4		
	DOWN	21	591		

*padj>0.05,LFC(1.5)

	Labeled by:	*Pdgfra*H2BeGFP	*Myh11*CreERT2;*Rosa26*tDtom
Fibroblasts	Trophocytes	✓	✗
	PDGFRAloCD55hi	✓	✗
	PDGFRAloCD55lo	✓	✗
	SEMF	✓	✓
Smooth muscle	LPM	✗	✓
	MM	✗	✓

Fig. S1. Growth requirements in irradiated organoids and molecular characteristics of isolated small intestinal stromal cell types following ISC loss.

A) Representative images of organoid EdU uptake (left) and quantitation (right: percent EdU^+ buds/organoid per condition, n $\geq$25 organoids/sample, mean $\pm$SEM). Scale bar, 50 μm. Significance was determined by one-way ANOVA followed by Tukey's multiple comparisons test and significance reported relative to unirradiated + BMPi (**p <0.01,****p <0.0001, n $\geq$3 independent organoid cultures).

B) qRT-PCR analysis of organoids grown in 1% RSPO with or without rNOG in untreated or 4 Gy irradiated conditions. Transcript levels are reported relative to untreated organoids without BMPi (mean $\pm$SEM). Significance was determined by one-way ANOVA followed by Tukey's multiple comparisons test. *p <0.05, ***p <0.001, ****p <0.0001.

C) Principal Component analysis of duplicate RNA profiles of SEMFs, $PDGFRA^{lo}$ fibroblasts, and blood (BECs) and lymphatic (LECs) endothelial cells in control, Diphtheria toxin-treated *Lgr5*^{Dtr} mice, and irradiated conditions. SEMFs and $PDGFRA^{lo}$ cells are substantially different from each other and from BECs or LECs, which cluster closely. Top right table: Cell marker expression from bulk RNA reveals consistency in cell-type isolation across conditions. Bottom right table: Numbers of differentially expressed genes (DEGs) significantly UP- or DOWN- regulated in ISC loss conditions compared to controls (P_{adj} >0.05, $\log_2$ |fold-change| >1.5, full list in Table S1).

D) Mouse lines used to isolate PDGFRA versus smooth muscle populations in scRNA sequencing experiments (black checkmark=labeled, red cross=not labeled).

E) Volcano plots show few overall changes in BECs compared to LECs after irradiation (P_{adj} >0.05, $\log_2$ |fold-change| >1.5).

F) Relative expression of select secreted niche factor mRNAs in indicated mesenchymal populations across conditions. The heatmap is prepared from normalized values for RPKM averaged from duplicate RNA-seq libraries of each cell type and condition (numbers on right represent the largest RPKM value for gene).

G) Overlapping DEGs in each indicated population from scRNA-seq analysis (full lists in Table S1, red: UP in treatment, blue: DOWN in treatment, Padj<0.05, absLFC=1).

Development • Supplementary information

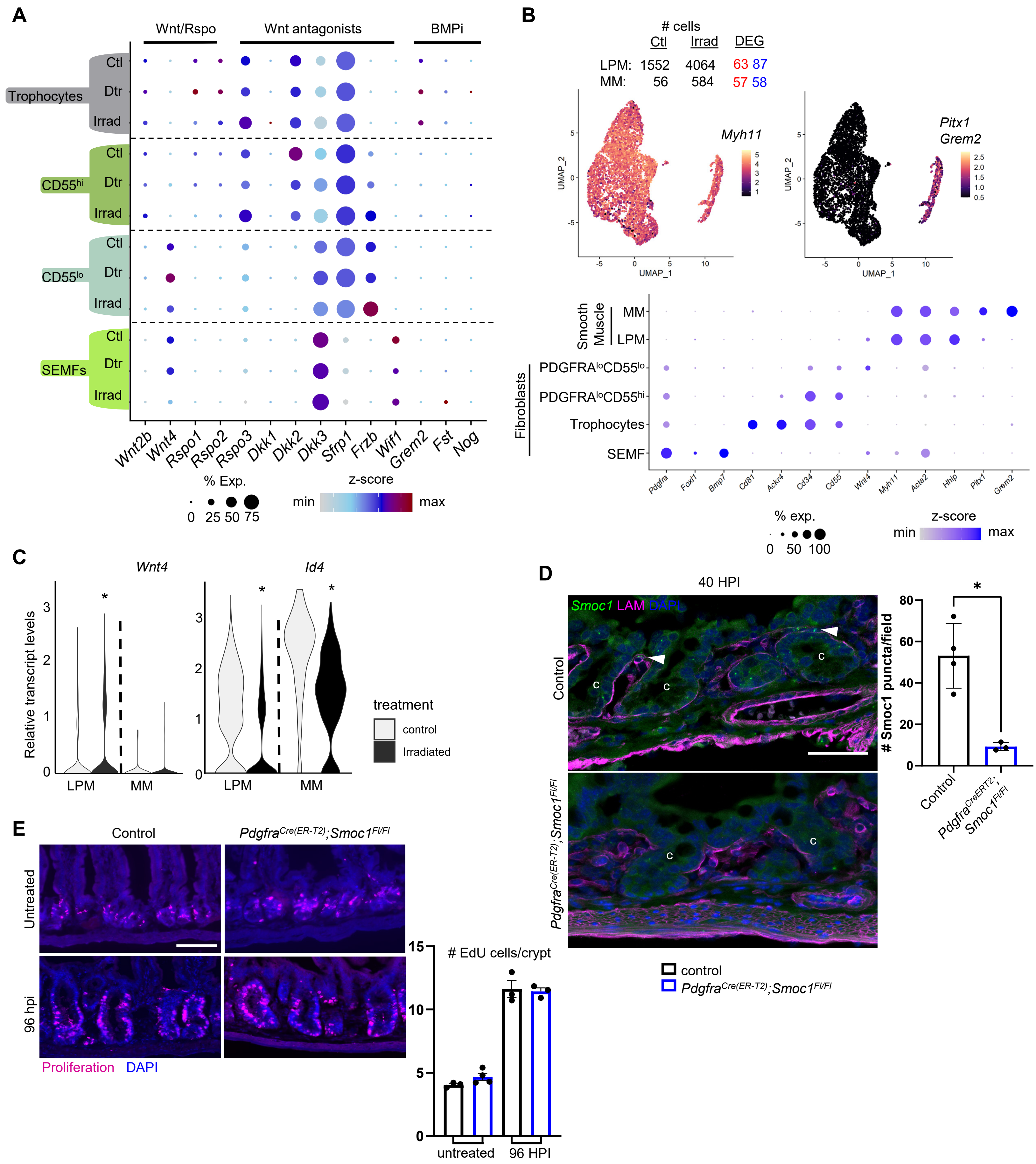

Development • Supplementary information

Fig. S2. scRNA sequencing analysis of growth factors across small intestinal stromal cell types following ISC loss and phenotypic analysis of *Pdgfra*$^{Cre(ER-T2)}$*;Smoc1*$^{Fl/Fl}$ mice.

A) scRNA-seq data on expression of positive and negative niche factors across treatments from integrated *Pdgfra*^{H2BeGFP} datasets. Circle sizes: percentage of cells expressing gene, fill colors: scaled average expression.

B) Both lamina propria myocytes (LPM) and muscularis mucosae (MM) express high levels of smooth muscle *Myh11*, while *Pitx2* and *Grem2* are MM-specific markers in integrated data from untreated and irradiated samples (see Methods, cell numbers listed above plot). DEG numbers in each irradiated population are compared to untreated mice (P_{adj} >0.05, log_2 |fold-change| >1.5; red=UP in treatment, blue=DOWN in treatment). Below: Dotplot of cell-specific markers generated from integrated data of untreated, irradiated, and Lgr5-Dtr samples of PDGFRA and smooth muscle scRNA libraries.

C) Violinplots show increased *Wnt4* in the LPM and reduced *Id4* in both smooth muscle populations after irradiation (Wilcoxon, *p<0.05).

D) In situ hybridization of *Smoc1* in control versus *Pdgfra*$^{Cre(ER-T2)}$*;Smoc1*$^{Fl/Fl}$ mice shows reduced mesenchymal *Smoc1*$^{+}$ puncta in the conditional knockout mice at 40 hours post irradiation (n=3, $\geq$10 fields/sample, mean $\pm$SEM). Scale bar, 50 μm.

E) Comparable cell proliferation (BrdU uptake, magenta) in untreated and 96 h post-irradiation control and *Pdgfra*$^{Cre(ER-T2)}$*;Smoc1*$^{Fl/Fl}$ animals (n =3 each, $\geq$ 40 crypts/sample, mean $\pm$SEM). Scale bar, 100 μm.

Development • Supplementary information

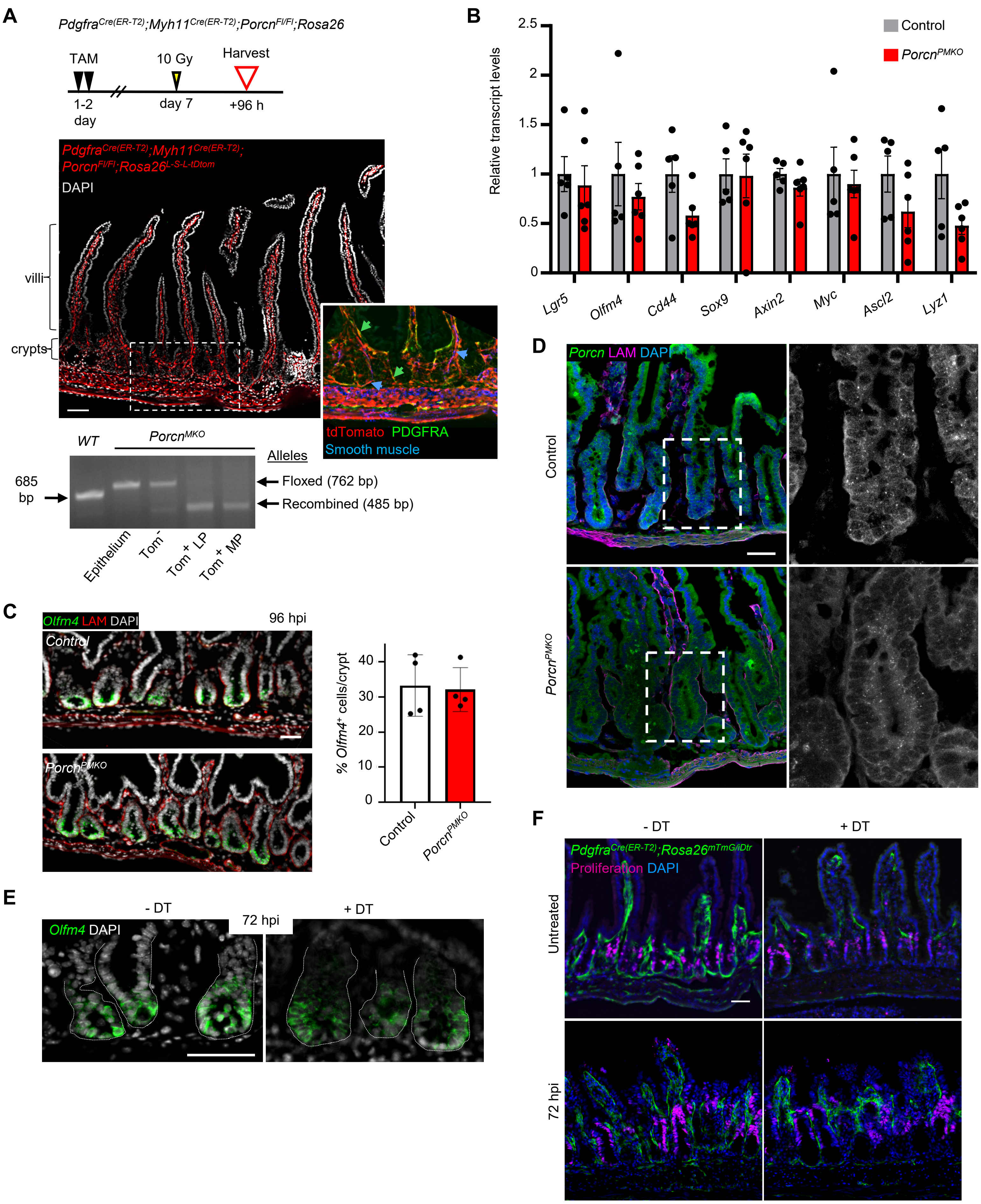

Development • Supplementary information

Fig. S3. ISCs regenerate independent of Wnt signaling from smooth muscle or SEMFs and phenotypic analysis of *Pdgfra*$^{Cre(ER\text{-}T2)}$*;Rosa26*$^{mTmG/iDtr}$ mice.

A) Cre activation was measured in *Pdgfra*$^{Cre(ER\text{-}T2)}$*;Myh11*$^{Cre(ER\text{-}T2)}$*;Porcn*$^{Fl/y}$*;Rosa26*^{tDtom} (*Porcn*^{PMKO}) mice by tDtom fluorescence, which overlapped with IHC staining for PDGFRA and ACTA2 (inset, PDGFRA$^+$tDtom$^+$ cells in green, ACTA2$^+$tDtom$^+$ smooth-muscle cells in blue). PCR genotyping of FACS-sorted tDtom$^+$ lamina propria and muscularis propria cells indicated recombination of the *Porcn* locus.

B) qRT-PCR analysis of ISC marker genes from crypts isolated from *Porcn*^{PMKO} mice. Transcript levels are represented relative to control crypts (mean $\pm$SEM). Significance, absent for all tested markers, was determined using unpaired two tailed t-test.

C) ISC regeneration 96 h post-irradiation was unperturbed in *Porcn*^{PMKO} mice (n=4, $\geq$25 crypts/sample, mean $\pm$SEM). Significance was determined using unpaired two tailed t test.

D) Representative image of *Porcn* in situ hybridization of control versus *Porcn*^{PMKO} small intestine at 96 hours post irradiation shows little expression in the mesenchyme compared to the epithelium in either condition (n=3/condition). Scale bar, 50 μm.

E) Representative image of *Olfm4* in situ hybridization at 72 h after irradiation of *Pdgfra*$^{Cre(ER\text{-}T2)}$*;Rosa26*$^{mTmG/iDtr}$ mice injected with saline (-DT) or Diphtheria toxin (+D).

F) Representative image of cell proliferation (EdU+ staining) in -DT or +DT *Pdgfra*$^{Cre(ER\text{-}T2)}$*;Rosa26*$^{mTmG/iDtr}$ mice in indicated conditions.

Development • Supplementary information

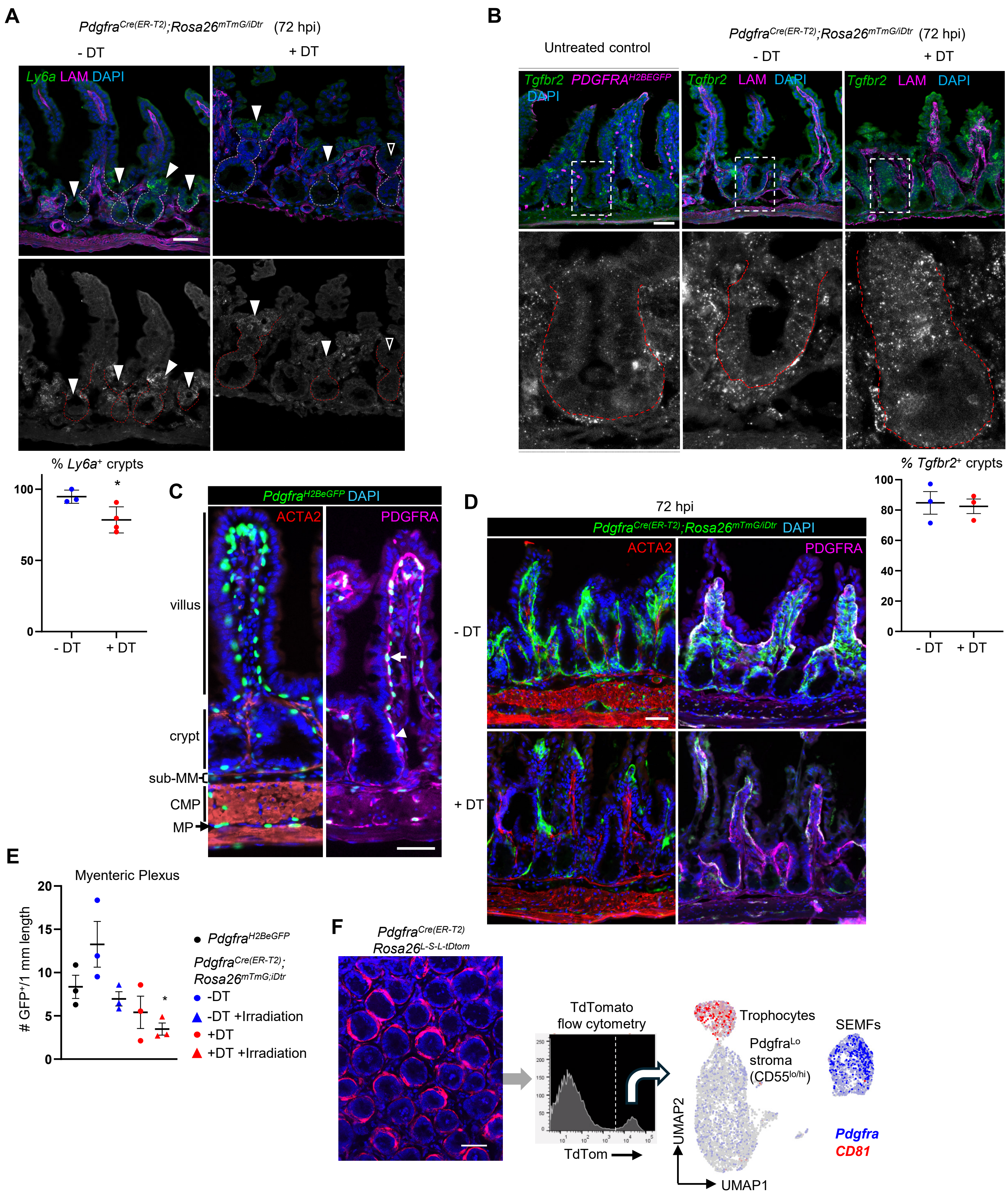

Development • Supplementary information

Fig. S4. Phenotypic analysis of *Pdgfra*^{CreERT2}*;Rosa26*$^{mtmg/iDtr}$ mice and scRNA sequencing analysis of *Pdgfra*$^{Cre(ER-T2)}$ tDtom^{+} cells.

A) In situ hybridization of *Ly6a* at 72 h after irradiation in *Pdgfra*$^{Cre(ER-T2)}$*;Rosa26*$^{mTmG/iDtr}$ mice injected with saline (-DT) or Diphtheria toxin (+DT, n=3, $\geq$40 crypts/sample, mean $\pm$SEM). Significance was determined using unpaired two tailed t-test, *p <0.05.

B) In situ hybridization of *Tgfbr2* in control versus 72 h after irradiation in *Pdgfra*$^{Cre(ER-T2)}$*;Rosa26*$^{mTmG/iDtr}$ mice -/+ DT (n=3, $\geq$40 crypts/sample, mean $\pm$SEM). No significance was found using unpaired two tailed t-test.

C) Representative image of untreated *Pdgfra*^{H2BeGFP} ileum stained for ACTA2, which labels muscularis mucosae (MM) and the external muscularis propria; and PDGFRA, which labels PDGFRA^{hi} SEMFs in the crypt (arrowhead) and villi (arrow).

D) Representative images of ACTA2- and PDGFRA-stained *Pdgfra*$^{Cre(ER-T2)}$*;Rosa26*$^{mTmG/iDtr}$mice at 72 hours post irradiation -/+ DT.

E) Quantification of numbers of GFP^{+} cells in the myenteric plexus of *Pdgfra*^{H2BeGFP} versus non-DT and DT-injected *Pdgfra*^{CreERT2}*;Rosa26*$^{mtmg/idtr}$ mice (>4mm length/sample, n=3/group, mean $\pm$SEM). Significance was determined using unpaired two tailed t-test between -/+ DT-injected *Pdgfra*$^{Cre(ER-T2)}$*;Rosa26*$^{mtmg/iDtr}$ mice within conditions. *p<0.05.

F) Whole-mount of *Pdgfra*$^{Cre(ER-T2)}$*;Rosa26*^{tDtom} mouse small intestine imaged from the serosal side 1 week after tamoxifen injection, showing tDtom^{+} SEMFs around crypts. Representative FACS histogram of tDtom^{+} cells isolated for scRNA-seq. Feature plot from scRNA-seq data reveals that labeled populations included trophocytes, other *Pdgfra*^{lo} stroma, and SEMFs. All scale bars, 50 μm.

Development • Supplementary information

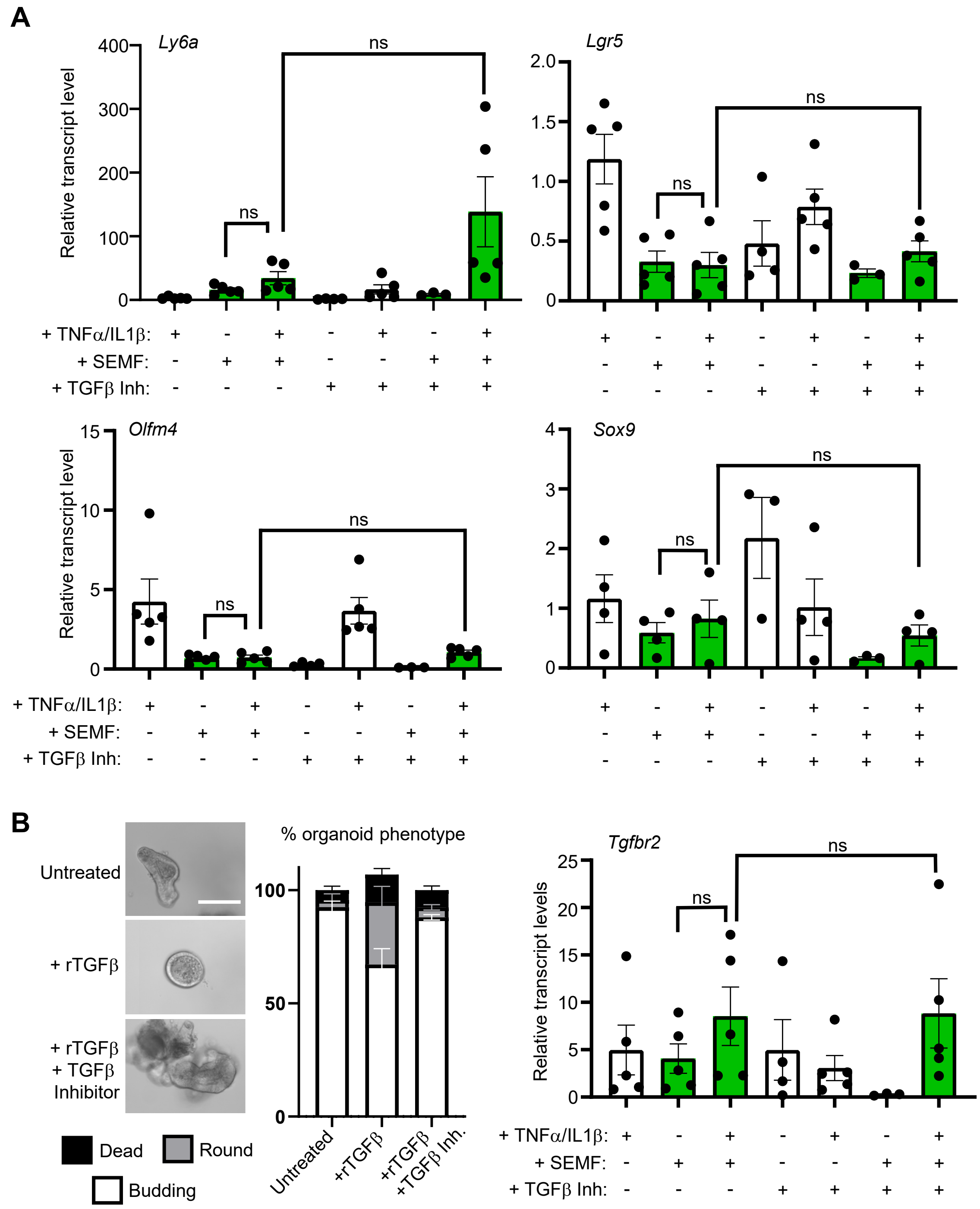

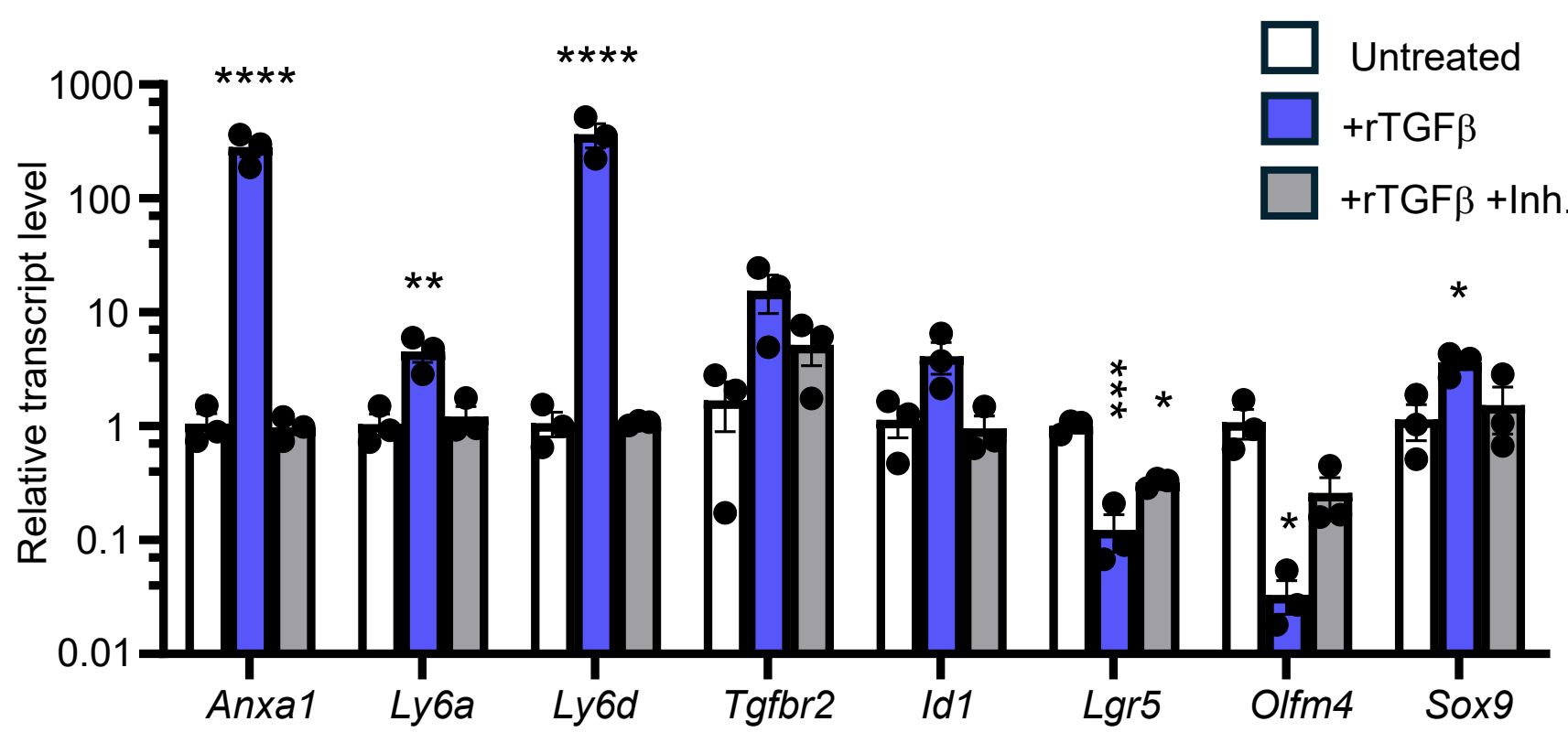

C

Genes	Forward Primer	Reverse Primer
Angptl4	CATCCTGGGACGAGATGAACT	TGACAAGCGTTACCACAGGC
Anxa1	GGAGAAGGGGACAGACGTG	TGGCACACTTCACGATGGTT
Ascl2	GAGCATGGAAGCACACCTTG	CTGCTACGAGTTCTGGTGCG
Axin2	TGACTCTCCTTCCAGATCCC	TGCCCACACTAGGGTGACA
Cd44	TGCCTCAACTGTGCACTCAA	GTTCTGGGCTTCTTGCCTCT
Ereg	TGTACCGCCTTAGTTCAGATGG	AGAAGTGCTCACATCGCAGA
Id1	GAGGCGGCATGTGTTCCA	CTGGAGGCTGAAAGGTGGAG
Igf1	CAACTCCCAGCTGTGCAATT	GCCGAGGTGAACACAAAACT
Lgr5	CCTACTCGAAGACTTACCCAGT	GCATTGGGGTGAATGATAGCA
Ly6a	CCTTCTCTGAGGATGGACACT	ACTTTCCTTGTTTGAGAATCCAC
Ly6d	AAAACCGTCACCTCAGTGGA	GACCAGCCTCTCGTTGCATA
Lyz1	GAGACCGAAGCACCGACTATG	CGGTTTTGACATTGTGTTCGC
Myc	CACATTGCCTTGCGGAAGAAG	GGTATTCCAGTGGACTCCAGAA
Olfm4	CAGCCACTTTCCAATTTCACTG	GCTGGACATACTCCTTCACCTTA
Smoc1	CGGCCCCAGGTTCCTAATAAG	GCACTTGGCTCTCTGGTACTC
Sox9	CCGGCATGAGTGAGGTGCAC	CTTTGCCAGCTTGCACGTCGG
Tgfb1	CTCCCGTGGCTTCTAGTGC	GCCTTAGTTTGGACAGGATCTG
Tgfbr2	GCGAGGAGTAAAGGCGCA	GCTTCCATTTCCACATCCGAC
Wnt5a	TGACAATCAGGAGGCGTGAG	GGGCGTGATTGTGCAAAAGA

Development • Supplementary information

Fig. S5. Organoid co-culture and treatment analysis.

A) qRT-PCR analysis of organoids collected from indicated conditions (n $\geq$3-5 independent organoid cultures, mean $\pm$SEM). Significance was determined by one-way ANOVA followed by Tukey's multiple comparisons test. No significance was found in indicated comparisons determined by one-way ANOVA followed by Tukey's multiple comparisons test.

B) Representative images of organoids cultured in indicated conditions and quantified in the graph shows that TGFβ inhibitor (Ly2109761) reduces TGFβ-induced rounding phenotype (n=3 independent organoid cultures, mean $\pm$SEM). Right: qRT-PCR of *Tgbfr2* in indicated conditions. Below: qRT-PCR of regenerative and stem cell markers shows TGFβ inhibitor as effective in blocking TGFβ-mediated organoid molecular signatures. Significance was determined by one-way ANOVA followed by Tukey's multiple comparisons test and reported relative to untreated at ****p <0.0001,***p <0.001, **p <0.01, *p <0.05 (n=3 independent organoid cultures, mean $\pm$SEM).

C) List of mouse primer sequences (forward and reverse) used for qRT-PCR analysis in this study.

Table S1. Differentially expressed genes (DEG) in distinct mesenchymal cell types following intestinal stem cell loss.

Available for download at
https://journals.biologists.com/dev/article-lookup/doi/10.1242/dev.205629#supplementary-data

Development • Supplementary information
