# Supplementary material for: Subepithelial myofibroblasts enhance stem cell regeneration after intestinal epithelial damage
Source: Development. 2026 Sep 29;153(18):dev205629. doi: 10.1242/dev.205629 (PMC13677008; doi:10.1242/dev.205629)
Supplement: Supplementary file 2 [file develop-153-205629_review_history.pdf]

Subepithelial myofibroblasts enhance stem cell regeneration after intestinal epithelial damage

Neil McCarthy, Camila Mendez Jimenez, Guodong Tie, Gizelle McCarthy, Shariq Madha-Krause, Ruiyang He and Ramesh A. Shivdasani
DOI: 10.1242/dev.205629

Editor: James Wells

Review timeline

Original submission:	2 March 2026
Editorial decision:	23 March 2026
First revision received:	31 July 2026
Accepted:	20 August 2026

Original submission

First decision letter

MS ID#: dev.205629

MS TITLE: Subepithelial myofibroblasts enhance stem cell regeneration after intestinal epithelial damage

AUTHORS: Neil McCarthy; Guodong Tie; Gizelle McCarthy; Shariq Madha-Krause; Ruiyang He; Ramesh Shivdasani

Dear Dr McCarthy,

I have now received all the referees' reports on the above manuscript, and have reached a decision. The referees' comments are appended below.

As you will see, the referees express considerable interest in your work, but have several significant criticisms and recommend a substantial revision of your manuscript before we can consider publication. If you are able to revise the manuscript along the lines suggested, which may involve further experiments, I will be happy to receive a revised version of the manuscript. Your revised paper will be re-reviewed by one or more of the original referees, and acceptance of your manuscript will depend on your addressing satisfactorily the reviewers' major concerns. Please also note that Development will normally permit only one round of major revision. If it would be helpful, you are welcome to contact us to discuss your revision in greater detail. Please send us a point-by-point response indicating your plans for addressing the referees' comments, and we will look over this and provide further guidance.

Please attend to all of the reviewers' comments and ensure that you upload both a 'clean' version of your Word file, along with a highlighted version clearly showing where you have made changes in the revised manuscript. Please avoid using 'Tracked changes' in Word files as these are lost in PDF conversion. I should be grateful if you would also provide a point-by-point response detailing how you have dealt with the points raised by the reviewers in the 'Response to Reviewers' box. If you do not agree with any of their criticisms or suggestions please explain clearly why this is so.

Reviewer 1

© 2026. Published by The Company of Biologists under the terms of the Creative Commons Attribution License (https://creativecommons.org/licenses/by/4.0/).

In this manuscript, McCarthy et al. examined the role of myofibroblasts in driving intestinal regeneration. By bulk and single cell RNA sequencing of stromal populations following whole body irradiation and ISC ablation, the authors found significant changes in expression of stem cell niche factors in myofibroblasts, whereas BMP and Wnt singlaing factors were relatively unchanged in Pdgfra low fibroblasts. Conditional deletion of Porcupine in Pdgfra expressing cells and smooth muscle cells did not impair ISC homeostasis and regeneration. Similarly, deletion of Smoc1, a BMP inhibitor had no significant effects on ISC maintenance in vivo. The authors also show that ablation of Pdgfra expressing cells was also insufficient to impact crypt regeneration in vivo but in vitro myofibroblasts phenocopied the effects of Tgfb or BMP treatment in organoid cocultures.

This manuscript represents an attempt to profile and functionally characterize the changes occurring in mesenchymal cells that contribute to ISC regeneration in vivo, which is a key question in the ISC field. Overall, the methodological approaches are sound and the results are clearly presented. However, the findings themselves provide limited new insight compared to what is already known in the field. The authors highlight the involvement of Smoc1 and SEMF as potential mediators of intestinal regeneration but fall short of showing convincing functional in vivo data of their importance to regeneration. Although the in vivo findings are negative (absence of a phenotype), the work demonstrates the high degree of robustness of the mesenchymal compartment in the gut, which would be useful to report but it's not clear that Development would be the best venue for this.

Specific comment:
In Figure 4G, the authors show that inflammatory cytokines like IL1b and Tnfa may drive expression of Tgfb and Smoc1 in fibroblasts. This is potentially the most interesting finding of the study but the analysis is limited. The manuscript would greatly benefit by characterizing this observation more thoroughly. Are Tgfb and Smoc1 the only genes affected ? Can SEMF cultures treated with cytokines further enhance organoid regenerative markers?

Reviewer 2

SUMMARY OF THE ADVANCE MADE IN THIS PAPER AND ITS POTENTIAL SIGNIFICANCE TO THE FIELD

McCarthy et al investigate the role of mesenchymal cells in intestinal regeneration using organoid cultures and two in vivo models, gamma irradiation and intestinal stem cell depletion. They identify SEMFs as the fibroblast population showing the most abundant transcriptional changes. However, neither depletion of SEMFs nor in vivo modulation of SEMF-specific WNT- or BMP-related pathways affects tissue regeneration. Instead, the authors suggest that Tgfb produced by SEMFs in response to inflammatory signals could be responsible for their pro-regenerative functions. This is an interesting study on the role of SEMFs in stem cell properties during repair that increases our understanding of the functions of individual fibroblast populations in the intestine.

SUGGESTIONS TO AUTHORS

Major
I have some major concerns related to PDGFRaCreERT2 mouse specificity and efficiency, and the proposed mechanism connecting inflammation, SEMF-specific Tgfb, and intestinal regeneration.
- The link between inflammation, Tgfb production by SEMFs, and intestinal organoid properties is weak. Inflammatory induction of SEMFs leads to a small upregulation of Smoc1 and Tgfb1, but the authors should explore its role in organoid cultures. In addition, one would expect local inflammation to induce an inflammatory gene expression in fibroblasts, including SEMFs. However, this is not evident in bulk or single-cell RNA sequencing. This should be commented on and discussed. Moreover, whether the effect of SEMFs - alone or induced with inflammatory factors - is Tgfb1-dependent should be shown in the organoid cultures, e.g., through inhibition of Tgfb signaling. The lack of in vivo evidence of pathophysiological significance similar to previous experiments for Porcn and Smoc1 should be discussed, especially since Smoc1 showed some in vitro effects, but was dispensable in vivo.
- PDGFRaCreERT2- mediated deletion of SEMFs should be better characterized. What are the exact percentages of fibroblast subpopulations targeted by this line? What is the percentage that is depleted per subpopulation? Supplementary Figure 1G is not clear; it could indicate PDGFRa+ cells

© 2026. Published by The Company of Biologists under the terms of the Creative Commons Attribution License (https://creativecommons.org/licenses/by/4.0/).

that were not targeted. Beyond proliferation and Olfm4+ crypts, is there any difference in regenerative gene expression (e.g. Ly6a, Ly6d)?
- Page 6, line 1-3, Fig. 3A: tdTomato expression does not necessarily mean efficient deletion of Prcn in all cells, as the locus is different. The levels of deletion should be tested at the RNA or protein level, preferably in individual subpopulations. Efficient deletion of Smoc1 in vivo should also be similarly verified. These control experiments are necessary to be able to conclude about their cell-specific functions.

Minor:
- In Fig. 1B,C quantifications, the significance represented by letters is not clear.
- Page 5, line 11: Changes in growth/regenerative factors are also worth mentioning in the conclusion.

Reviewer 3

SUMMARY OF THE ADVANCE MADE IN THIS PAPER AND ITS POTENTIAL SIGNIFICANCE TO THE FIELD

The contribution of different non-epithelial cell types (i.e., immune, endothelial, fibroblast, smooth muscle) of the intestinal stem cell niche during intestinal regeneration are not well defined. This report by McCarthy et al investigates the growth factor responses of specific niche fibroblasts and smooth muscle cells during intestinal regeneration after mouse whole body irradiation and Lgr5 cell ablation. A major focus is on Wnt/R-Spo and BMP/TGFb signaling pathways that are essential for sustaining ISCs at homeostasis, but other growth factors pathways are also examined.

Isolation of PDGFRAhi SEMFs, PDGFRAlow (CD55low, CD55hi and trophocytes), blood and lymphatic endothelial cells, and smooth muscle cells from the lamina propria myocytes and muscularis mucosae was achieved using sophisticated fluorescent reporter mice and cell surface FACS sorting. Gene expression responses of different sub-epithelial cell populations to injury were assessed by bulk RNAseq and scRNAseq analysis. With the exception of SEMFs, only modest changes in growth factor gene expression were observed in most cell populations. Under homeostasis, SEMFs express BMPs and regulate differentiation as cells exit crypts. However, upon injury, SEMFs increased expression of non-canonical Wnt5a and reduced expression of Wnt4 and Wif1 but also increased gene expression of Tgfb1 and a putative BMPi Smoc1 and decreased expression of numerous BMP ligands. Of note, other regenerative factors Angptl4, Igf, Areg, Ereg and Nrg1 were also increased. These findings suggest that SEMFs may promote intestinal regeneration through changes in growth factor responses, in part through increased Wnt and BMPi activity. The requirement for Wnt activity and Smoc1 were addressed through in vivo loss of function studies but both activities were dispensable during regeneration.

Finally, in vivo SEMF ablation studies demonstrated minimal change in both homeostasis and regenerative response. Because of potential limitations of the SEMF ablation studies, organoids were co-cultured with SEMFs and showed a complex response involving TGFb and BMP signaling. Interestingly, inflammatory cytokine treatment of SEMFs increased TGFb1 and Smoc1 expression. Overall, the significance to the field is a comprehensive analysis of the growth factor responses of different fibroblast and smooth muscle cell populations of the intestinal niche during injury. Only SEMFs showed demonstrable changes in growth factor gene expression particularly within BMP/TGFb signaling that may promote regeneration. However, SEMFs were dispensable which highlights the complex cellular nature of responses in the intestinal stem cell niche signaling that maybe co-ordinated in part by inflammatory signals.

SUGGESTIONS TO AUTHORS

With so many cell populations to be discussed and with a primary focus on SEMFs, the current text particularly associated with bulk RNAseq and scRNAseq is difficult to follow. It might be easier to focus on all scRNAseq data in primary figure 2 and present bulk RNAseq data in an associated supplementary figure. In addition, it might be clearer to address the responses of all the other cell types first and then focus on the SEMFs.

© 2026. Published by The Company of Biologists under the terms of the Creative Commons Attribution License (https://creativecommons.org/licenses/by/4.0/).

In Figure 1, it would be helpful to state that crypt-derived organoids were grown in ENR with varying concentrations of R-Spondin CM (even though stated in methods) which allows integration of endogenous Wnt activity from organoids.

In Figure 2A, the graphical depiction of all cell types is very helpful. The crosses presumably depict ISC loss is accurate for Lgr5 ablation but is misleading for whole body irradiation model.

The description of CD81+ trophocytes is only outlined in Figure 2A and then defined more in the scRNAseq data in Fig. 2C/G. In supplement, it might be helpful to have a table outlining the sorting strategies and markers for all fibroblast and smooth muscle cell populations.

In Figure 3, a description of the experimental design for Tamoxifen treatment followed by irradiation in PdgfraCre(ER-T2); Myh11Cre(ER-T2); Prcnfl/fl conditional mutant (PrcnPMKO) mice is not provided.
In Fig 3E, no comparison for representative Olfm4 expression with control (not tamoxifen treated).
In Sup Fig 2E images for BrdU labeling are compared with control but quantification is not provided.

The authors state, "Although PdgfraCre(ER-T2) is expressed in all PDGFRA+ populations (Supplemental Figure 2D), most of these are SEMFs (Kraiczy et al., 2023)" and is used for Prcn and Smoc1 deletion and the SEMF ablation studies. Prcn recombination in tdTom+ LPs and MPs and Smoc1 recombination in tdTom+ cells is observed. But the authors also state "While many PDGFRAlo cells might escape Prcn deletion, the findings indicate that Wnt secretion from smooth muscle or SEMFs is largely dispensable for ISC homeostasis and regeneration". Similarly, the authors question the efficiency of PDGFRACreER to cause DTR-mediated ablation in all PDGRFAlo cells. The authors state "Therefore, the lack of overt post-radiation defects in these experiments could reflect absence of SEMF requirement in ISC regeneration or insufficient SEMF ablation compounded by partial recovery of ablated cells". Indeed, there appears to be significant mGFP+ cells within villus compartment in Fig 4 B which presumably are PDGFRAlo cells. In addition, the recovery of PDGFRA+ cells is somewhat concerning. It is acknowledged that the authors are using state of art approaches to isolate PDGFRA+ cells but the deficiencies associated with PDGFRACreER weaken the findings. Is there a way to strengthen the approach and improve analysis through combining with PDGFRH2b-eGFP reporter, optimizing timing of DT treatment or combining with end-point FACS analysis/scRNAseq?

Throughout the manuscript, the graphical representation of statistical information differs - a similar format would make results easier to interpret.

Image magnification of Figs 3C, 4A, B and Sup 2E and F should be increased.

For Fig 4B and Sup Fig 2F, the graphs depict Olfm4+ crypts and EdU proliferation but the images associated with this data are not matched. Fig 4B shows Olfm4 expression +/- DT treatment but not irradiation whereas Sup Fig 2F shows EdU labeling +/- DT treatment followed by 72h post irradiation but not DT treatment alone.

Organoid co-cultures with SEMFs - SEMFs were cultured as monolayers prior to co-culture experiments. which may lead to an activated state prior to co-culture which might change their gene expression. Do untreated and cytokine treated cultured SEMFs express TGFb Smoc1 Wnt5a Igf1 at similar levels to baseline and after radiation in vivo?

First revision

Author response to reviewers' comments

Reviewer 1

General: In this manuscript, McCarthy et al. examined the role of myofibroblasts in driving

© 2026. Published by The Company of Biologists under the terms of the Creative Commons Attribution License (https://creativecommons.org/licenses/by/4.0/).

intestinal regeneration. By bulk and single cell RNA sequencing of stromal populations following whole body irradiation and ISC ablation, the authors found significant changes in expression of stem cell niche factors in myofibroblasts, whereas BMP and Wnt singlaing factors were relatively unchanged in Pdgfra low fibroblasts. Conditional deletion of Porcupine in Pdgfra expressing cells and smooth muscle cells did not impair ISC homeostasis and regeneration. Similarly, deletion of Smoc1, a BMP inhibitor had no significant effects on ISC maintenance in vivo. The authors also show that ablation of Pdgfra expressing cells was also insufficient to impact crypt regeneration in vivo but in vitro myofibroblasts phenocopied the effects of Tgfb or BMP treatment in organoid cocultures.

This manuscript represents an attempt to profile and functionally characterize the changes occurring in mesenchymal cells that contribute to ISC regeneration in vivo, which is a key question in the ISC field. Overall, the methodological approaches are sound and the results are clearly presented. However, the findings themselves provide limited new insight compared to what is already known in the field. The authors highlight the involvement of Smoc1 and SEMF as potential mediators of intestinal regeneration but fall short of showing convincing functional in vivo data of their importance to regeneration. Although the in vivo findings are negative (absence of a phenotype), the work demonstrates the high degree of robustness of the mesenchymal compartment in the gut, which would be useful to report but it's not clear that Development would be the best venue for this.

Response: We appreciate the Reviewer's recognition of complexities in the field, our sound methodologies and presentation, and strengths as well as weakness in our original manuscript. We believe that addressing the Reviewer's specific comments has improved the study and enhances its contribution to a challenging but important area of investigation.

1) In Figure 4G, the authors show that inflammatory cytokines like IL1b and Tnfa may drive expression of Tgfb and Smoc1 in fibroblasts. This is potentially the most interesting finding of the study but the analysis is limited. The manuscript would greatly benefit by characterizing this observation more thoroughly. Are Tgfb and Smoc1 the only genes affected ? Can SEMF cultures treated with cytokines further enhance organoid regenerative markers?

Response: We agree that the findings we originally presented in Fig. 4 warranted a deeper dive. As Reviewer 1 suggested, we interrogated additional features of SEMF responses to pro-inflammatory cytokines. We now show that IL1b and TNF1a indeed induce genes other than *Tgfb* and *Smoc1* in SEMFs, including those associated with regenerative activity, such as *Ereg* and *Angplt4*. Importantly, these genes are not activated by γ-irradiation; instead, they represent the SEMF response to local inflammation (new Fig. 4B). To address the link between inflammation and SEMF effects on epithelial regeneration, we co-cultured organoids with SEMFs in the presence or absence of IL1b and TNF1a, with appropriate controls. In the organoids we observed substantial induction of a portion of the epithelial regenerative program, including *Anxa1* and especially *Ly6d1* (Fig. 4C). A pharmacologic TGFb receptor inhibitor potently blocked untreated SEMF effects but not the regenerative program induced by IL1b/TNF1a. We thus conclude that the mesenchymal influence on ISC restoration has both TGFb-dependent and TGFb-independent arms. Together, these findings enhance and deepen what the Reviewer regarded as our most interesting finding and further improve our manuscript's fit for a special issue of Development dedicated to the theme of "The Extracellular Environment in Development, Regeneration and Stem Cells".

Reviewer 2

General: McCarthy et al investigate the role of mesenchymal cells in intestinal regeneration using organoid cultures and two in vivo models, gamma irradiation and intestinal stem cell depletion. They identify SEMFs as the fibroblast population showing the most abundant transcriptional

© 2026. Published by The Company of Biologists under the terms of the Creative Commons Attribution License (https://creativecommons.org/licenses/by/4.0/).

changes. However, neither depletion of SEMFs nor in vivo modulation of SEMF-specific WNT- or BMP-related pathways affects tissue regeneration. Instead, the authors suggest that Tgfb produced by SEMFs in response to inflammatory signals could be responsible for their pro-regenerative functions. This is an interesting study on the role of SEMFs in stem cell properties during repair that increases our understanding of the functions of individual fibroblast populations in the intestine.

I have some major concerns related to PDGFRaCreERT2 mouse specificity and efficiency, and the proposed mechanism connecting inflammation, SEMF-specific Tgfb, and intestinal regeneration.

Response: We thank the Reviewer for the positive feedback and for articulating specific concerns. The revised manuscript addresses each of the below critiques.

1) The link between inflammation, Tgfb production by SEMFs, and intestinal organoid properties is weak. Inflammatory induction of SEMFs leads to a small upregulation of Smoc1 and Tgfb1, but the authors should explore its role in organoid cultures. In addition, one would expect local inflammation to induce an inflammatory gene expression in fibroblasts, including SEMFs. However, this is not evident in bulk or single-cell RNA sequencing. This should be commented on and discussed. Moreover, whether the effect of SEMFs - alone or induced with inflammatory factors - is Tgfb1- dependent should be shown in the organoid cultures, e.g., through inhibition of Tgfb signaling. The lack of in vivo evidence of pathophysiological significance similar to previous experiments for Porcn and Smoc1 should be discussed, especially since Smoc1 showed some in vitro effects, but was dispensable in vivo.

Response: These concerns are well taken and we thank the Reviewer for suggesting experiments that yielded additional insight. On the Reviewer's recommendation, we addressed the link between SEMFs, inflammation, and TGFb production on organoid properties: we co-cultured organoids with SEMFs with or without pro-inflammatory cytokines IL1b and TNF1a and with or without a TGFb inhibitor. When co-cultured with cytokine-treated SEMFs (compared to untreated SEMFs), organoids activated regenerative markers *Anxa1* and especially *Ly6d1* (Fig. 4C). We then identified a concentration of a TGFb receptor inhibitor (LY2109761) that blocked TGFb- induced effects, including selected regenerative markers (new Fig. S5B). This concentration of LY2109761 blocked induction of regenerative genes by untreated SEMFs but not by cytokine- treated SEMFs (Fig. 4C). Therefore, factors other than TGFb underlie cytokine-induced SEMF assistance of ISC regeneration.

The revised manuscript discusses the requested points (within the confines of tight word limits) and explicitly states that singular loss of Smoc1 or Porcn and reduction of SEMFs likely don't impair ISC regeneration *in vivo* owing to robust tissue redundancies (Page 5, line 12; Page 5, line 31). Our *in vitro* studies are more revealing because the experimental design minimizes those redundancies and other variables. While our study focused on regenerative signals, a recent scRNA-seq study (PMID: 38569642) identified inflammatory changes in colonic SEMFs; our RNA-seq data also showed such changes in SEMFs after whole-body irradiation (Table S1; Page 8, line 1).

2) PDGFRaCreERT2- mediated deletion of SEMFs should be better characterized. What are the exact percentages of fibroblast subpopulations targeted by this line? What is the percentage that is depleted per subpopulation? Supplementary Figure 1G is not clear; it could indicate PDGFRa+ cells that were not targeted. Beyond proliferation and Olfm4+ crypts, is there any difference in regenerative gene expression (e.g. Ly6a, Ly6d)?

Response: This is a fair critique. We previously characterized *Pdgfra*^{CreERT2}-mediated tissue effects (PMID 37028407) but the Reviewer is right that additional information will better support our interpretations in the present manuscript. To quantify PDGFRA^{+} cell depletion, we counted GFP^{-} and GFP^{+} cells in *Pdgfra*^{CreERT2};*Rosa26*$^{mTmG/iDTR}$ mice, comparing the numbers to counts

© 2026. Published by The Company of Biologists under the terms of the Creative Commons Attribution License (https://creativecommons.org/licenses/by/4.0/).

in *Pdgfra*^{H2BeGFP} mice. Tamoxifen-treated *Pdgfra*^{CreERT2};*Rosa26*$^{mTmG/iDTR}$ mice mark all SEMFs and PDGFRA^{+} cells in the circumferential muscularis propria (CMP) but fewer PDGFRA^{lo}CD55^{lo} (villus) and CD55^{hi} (crypt) cells and even fewer sub-cryptal trophocytes. Diphtheria toxin treatment significantly reduced GFP+ cells numbers in each compartment except trophocytes (new Fig. 3C). On the Reviewer's recommendation, we also assessed other regeneration markers in DT-injected mice and observed a slight reduction in *Ly6a*-marked but similar numbers of *Tgfbr2*-labeled crypts (new Fig. S4A-B). In these regions, PDGFRA antibody labeling showed comparable numbers of peri-cryptal PDGFRA+ cells in DT-treated and untreated mice (Fig. 3D). As the revised manuscript clarifies, although SEMFs were substantially reduced, residual peri-cryptal cells could in principle account for persistent ISC regeneration. Unfortunately, this is the only existing mouse model to test SEMF requirements by cell ablation *in vivo*, and incidentally, additional DT injections to enhance ablation of PDGFRA^{+} cells caused severe morbidity, even in mice lacking the *Cre* allele (data not shown).

3) Page 6, line 1-3, Fig. 3A: tdTomato expression does not necessarily mean efficient deletion of Prcn in all cells, as the locus is different. The levels of deletion should be tested at the RNA or protein level, preferably in individual subpopulations. Efficient deletion of Smoc1 in vivo should also be similarly verified. These control experiments are necessary to be able to conclude about their cell- specific functions.

Response: This is a fair point, related to point #2 above. In response, we assessed *Smoc1* depletion by in situ hybridization, which showed a significant decrease at 40 h after ISC ablation, when levels are highest in control animals (new Fig. S2D). By contrast, *Porcn* in situ hybridization gave extremely low mesenchymal signal and higher epithelial signal (new Fig. S3D – mesenchymal cell RNA levels are also low in bulk RNA-seq). Low *Porcn* levels notwithstanding, genomic PCR confidently showed recombination of the floxed allele (Fig. S3A) in TdTom^{+} cells and not TdTom^{-} or epithelial cells.

Minor:
- In Fig. 1B,C quantifications, the significance represented by letters is not clear.

Response: We agree that the lettering schema was needlessly complicated. Throughout the revised manuscript, significance is now indicated by simple asterisks (and in one case # signs) that are annotated in the figure legends.

- Page 5, line 11: Changes in growth/regenerative factors are also worth mentioning in the conclusion.

Response: We appreciate this suggestion, which we have adopted (Page 4, line 28).

Reviewer 3

General: The contribution of different non-epithelial cell types (i.e., immune, endothelial, fibroblast, smooth muscle) of the intestinal stem cell niche during intestinal regeneration are not well defined. This report by McCarthy et al investigates the growth factor responses of specific niche fibroblasts and smooth muscle cells during intestinal regeneration after mouse whole body irradiation and Lgr5 cell ablation. A major focus is on Wnt/R-Spo and BMP/TGFb signaling pathways that are essential for sustaining ISCs at homeostasis, but other growth factors pathways are also examined.

Isolation of PDGFRAhi SEMFs, PDGFRAlow (CD55low, CD55hi and trophocytes), blood and lymphatic endothelial cells, and smooth muscle cells from the lamina propria myocytes and muscularis mucosae was achieved using sophisticated fluorescent reporter mice and cell surface FACS sorting.

© 2026. Published by The Company of Biologists under the terms of the Creative Commons Attribution License (https://creativecommons.org/licenses/by/4.0/).

Gene expression responses of different sub-epithelial cell populations to injury were assessed by bulk RNAseq and scRNAseq analysis. With the exception of SEMFs, only modest changes in growth factor gene expression were observed in most cell populations. Under homeostasis, SEMFs express BMPs and regulate differentiation as cells exit crypts. However, upon injury, SEMFs increased expression of non-canonical Wnt5a and reduced expression of Wnt4 and Wif1 but also increased gene expression of Tgfb1 and a putative BMPi Smoc1 and decreased expression of numerous BMP ligands. Of note, other regenerative factors Angptl4, Igf, Areg, Ereg and Nrg1 were also increased. These findings suggest that SEMFs may promote intestinal regeneration through changes in growth factor responses, in part through increased Wnt and BMPi activity. The requirement for Wnt activity and Smoc1 were addressed through in vivo loss of function studies but both activities were dispensable during regeneration.

Finally, in vivo SEMF ablation studies demonstrated minimal change in both homeostasis and regenerative response. Because of potential limitations of the SEMF ablation studies, organoids were co-cultured with SEMFs and showed a complex response involving TGFb and BMP signaling. Interestingly, inflammatory cytokine treatment of SEMFs increased TGFb1 and Smoc1 expression. Overall, the significance to the field is a comprehensive analysis of the growth factor responses of different fibroblast and smooth muscle cell populations of the intestinal niche during injury. Only SEMFs showed demonstrable changes in growth factor gene expression particularly within BMP/TGFb signaling that may promote regeneration. However, SEMFs were dispensable which highlights the complex cellular nature of responses in the intestinal stem cell niche signaling that maybe co-ordinated in part by inflammatory signals.

Response: We thank the Reviewer for a comprehensive appraisal of the study. The constructive and specific suggestions to improve the manuscript are much appreciated.

1. With so many cell populations to be discussed and with a primary focus on SEMFs, the current text particularly associated with bulk RNAseq and scRNAseq is difficult to follow. It might be easier to focus on all scRNAseq data in primary figure 2 and present bulk RNAseq data in an associated supplementary figure. In addition, it might be clearer to address the responses of all the other cell types first and then focus on the SEMFs.

Response: This is a helpful recommendation. The revised manuscript is reorganized to discuss non-SEMF cells first and, as the Reviewer suggested, bulk RNA analysis is moved to Fig. S1C-F.

2. In Figure 1, it would be helpful to state that crypt-derived organoids were grown in ENR with varying concentrations of R-Spondin CM (even though stated in methods) which allows integration of endogenous Wnt activity from organoids.

Response: Thank you for this suggestion, which we have implemented (Fig. 1A).

3. In Figure 2A, the graphical depiction of all cell types is very helpful. The crosses presumably depict ISC loss is accurate for Lgr5 ablation but is misleading for whole body irradiation model.

Response: Thank you for this helpful suggestion. The data are now presented in Fig. 1B with an altered schematic that depicts crypt cells affected by irradiation with red crosses and more precise DT-induced ablation of *Lgr5*$^{+}$ ISCs in black.

4. The description of CD81+ trophocytes is only outlined in Figure 2A and then defined more in the scRNAseq data in Fig. 2C/G. In supplement, it might be helpful to have a table outlining the sorting strategies and markers for all fibroblast and smooth muscle cell populations.

Response: This is a very good idea, thank you. New Fig. S1D outlines the sorting strategies for

© 2026. Published by The Company of Biologists under the terms of the Creative Commons Attribution License (https://creativecommons.org/licenses/by/4.0/).

FACS isolations and new Fig. S2B details molecular markers that distinguish fibroblast from various smooth muscle cell types.

5. In Figure 3, a description of the experimental design for Tamoxifen treatment followed by irradiation in PdgfraCre(ER-T2); Myh11Cre(ER-T2); Prcnfl/fl conditional mutant (PrcnPMKO) mice is not provided.

Response: Thank you for noting this omission. A schematic of the experimental design is now incorporated into Fig. S3A.

6. In Fig 3E, no comparison for representative Olfm4 expression with control (not tamoxifen treated). In Sup Fig 2E images for BrdU labeling are compared with control but quantification is not provided.

Response: Thank you for noting these deficiencies. The revised manuscript includes an image of Olfm4 expression in control samples (Fig. 2C) and $BrdU^{+}$ cells/crypt are quantified in $Pdgfra^{Cre}$;$Smoc1^{Fl/Fl}$ mice at 96 hpi (Fig. S2E).

7. The authors state, "Although PdgfraCre(ER-T2) is expressed in all PDGFRA+ populations (Supplemental Figure 2D), most of these are SEMFs (Kraiczy et al., 2023)" and is used for Prcn and Smoc1 deletion and the SEMF ablation studies. Prcn recombination in tdTom+ LPs and MPs and Smoc1 recombination in tdTom+ cells is observed. But the authors also state "While many PDGFRAlo cells might escape Prcn deletion, the findings indicate that Wnt secretion from smooth muscle or SEMFs is largely dispensable for ISC homeostasis and regeneration". Similarly, the authors question the efficiency of PDGFRACreER to cause DTR-mediated ablation in all PDGRFAlo cells. The authors state "Therefore, the lack of overt post-radiation defects in these experiments could reflect absence of SEMF requirement in ISC regeneration or insufficient SEMF ablation compounded by partial recovery of ablated cells". Indeed, there appears to be significant mGFP+ cells within villus compartment in Fig 4 B which presumably are PDGFRAlo cells. In addition, the recovery of PDGFRA+ cells is somewhat concerning. It is acknowledged that the authors are using state of art approaches to isolate PDGFRA+ cells but the deficiencies associated with PDGFRACreER weaken the findings. Is there a way to strengthen the approach and improve analysis through combining with PDGFRH2b-eGFP reporter, optimizing timing of DT treatment or combining with end-point FACS analysis/scRNAseq?

Response: These fair concerns echo point #2 from Reviewer 2. Both *Pdgfra*^{H2BeGFP} *and* *Pdgfra*^{CreERT2} being null alleles, compound heterozygotes are inviable (PMID: 10903171), so the ideal experiment the Reviewer suggests is unfortunately not feasible. Instead, we defined more accurately the cells that express *Pdgfra*^{CreERT2} by their anatomical location in comparison to GFP+ cells in *Pdgfra*^{H2BeGFP} mice (new Fig. 3C). Tamoxifen-treated *Pdgfra*^{CreERT2};*Rosa26*$^{mTmG/iDTR}$ mice mark all SEMFs and $PDGFRA^{+}$ cells in the circumferential muscularis propria (CMP) but fewer $PDGFRA^{lo}CD55^{lo}$ (villus) and $CD55^{hi}$ (crypt) cells and even fewer trophocytes. DT treatment reduced GFP+ cells numbers in each compartment except trophocytes (new Fig. 3C). However, PDGFRA antibody labeling showed comparable numbers of peri-cryptal PDGFRA+ cells in DT-treated and untreated mice (Fig. 3D). As the revised manuscript clarifies, although SEMFs were substantially reduced, residual peri-cryptal cells could in principle account for persistent ISC regeneration. Unfortunately, this is the only existing mouse model to test SEMF requirements by cell ablation *in vivo*, and incidentally, additional DT injections to enhance ablation of $PDGFRA^{+}$ cells caused severe morbidity, even in mice lacking the *Cre* allele (data not shown).

© 2026. Published by The Company of Biologists under the terms of the Creative Commons Attribution License (https://creativecommons.org/licenses/by/4.0/).

8. Throughout the manuscript, the graphical representation of statistical information differs - a similar format would make results easier to interpret.

Response: This is a fair point. Throughout the revised manuscript, statistical information is now conveyed more simply and consistently.

9. Image magnification of Figs 3C, 4A, B and Sup 2E and F should be increased.

Response: Thank you for this suggestion. Magnifications of these images are increased in the revised manuscript (now Fig. S3C, Fig. 3A, 3B, and Fig. S2E and S3F, respectively).

10. For Fig 4B and Sup Fig 2F, the graphs depict Olfm4+ crypts and EdU proliferation but the images associated with this data are not matched. Fig 4B shows Olfm4 expression +/- DT treatment but not irradiation whereas Sup Fig 2F shows EdU labeling +/- DT treatment followed by 72h post irradiation but not DT treatment alone.

Response: Thank you for noting these omissions. The revised manuscript includes images of *Olfm4* and EdU staining in control and irradiated Pdgfra^{CreERT2} mice with and without DT treatment (Fig. S3E-F).

11. Organoid co-cultures with SEMFs - SEMFs were cultured as monolayers prior to co-culture experiments. which may lead to an activated state prior to co-culture which might change their gene expression. Do untreated and cytokine treated cultured SEMFs express TGFb Smoc1 Wnt5a Igf1 at similar levels to baseline and after radiation in vivo?

Response: We appreciate this insightful comment. Monolayer SEMF cultures might indeed harbor a baseline "activated" state and altered gene activity prior to co-culture. Evaluating that transcriptional modulation is an important goal for the field, but rigorous and comprehensive assessment of distinct microenvironments falls outside the scope of our Brief Report. The revised manuscript nevertheless concludes with transparent acknowledgment that organoid co-cultures don't fully replicate *in vivo* conditions.

Second decision letter

MS ID#: dev.205629R1

MS TITLE: Subepithelial myofibroblasts enhance stem cell regeneration after intestinal epithelial damage

AUTHORS: Neil McCarthy; Camila Mendez Jimenez; Guodong Tie; Gizelle McCarthy; Shariq Madha-Krause; Ruiyang He; Ramesh Shivdasani

Dear Dr McCarthy,

I am happy to tell you that your manuscript has been accepted for publication in Development, pending our standard publication integrity checks.

It was accepted on 20 Aug 2026. Where referee reports on this version are available, they are appended below. As you will see below, one of the reviewers suggested additional experiments that were not in the initial reviews. Having read over the critiques and your revisions, I feel that your revised manuscript addressed the major concerns of the initial reviews.

© 2026. Published by The Company of Biologists under the terms of the Creative Commons Attribution License (https://creativecommons.org/licenses/by/4.0/).

Reviewer 1

The revised manuscript provides new data showing that treatment of SEMFs with IL1b and Tnfa induces 2 additional genes potentially associated with intestinal regeneration. In total 4 genes are found differentially expressed including Tgfb, Smoc1, Ereg and Angplt4. In addition, the authors demonstrate that treatment of co-cultures with IL1b and Tnfa enhances induction of Anxa1 and Ly6d1, which can be interpreted as a regenerative response.

Although these additions technically address my specific comments and somewhat improve the manuscript, it is also my assessment that the new data represents a missed opportunity. Considering the thorough analysis of the transcriptional changes associated with ISC depletion in vivo presented in Figure 1, it is surprising that the authors did not perform a simple bulk RNAseq of IL1b and TNFa treated SEMF to reveal a more comprehensive picture of the changes in gene expression induced by these factors. Even though I agree that these experiments were not explicitly requested in my initial review, the superficial analysis in Figure 4 contrasts the first part of the manuscript.

Similar comments may be made with regards to the coculture experiments. 2 genes were found upregulated. An unbiased approach using bulk RNAseq to identify the responsive genes to ILb and Tnf would have been much more informative and could have been easily done. Anxa1 and Ly6d1 are potentially associated with a regenerative response, but without any effects shown on organoid growth it is rather weak evidence.

Additional comment:
How can the authors be certain that Anax1 and Ly6d1 are upregulated in epithelial cells when the RNA extraction was performed from co-cultures including fibroblasts and epithelial cells?

Reviewer 2

SUMMARY OF THE ADVANCE MADE IN THIS PAPER AND ITS POTENTIAL SIGNIFICANCE TO THE FIELD

The paper by McCarthy et al sheds light on the role of distinct intestinal fibroblast populations during intestinal regeneration. The focus on SEMFs and their functions is especially important, as this population is not considered part of the homeostatic intestinal stem cell niche. The role of inflammatory signals in this process is also noteworthy, adding to the complexity of the tissue restoration mechanisms.

SUGGESTIONS TO AUTHORS

The authors have performed additional experiments, expanded the discussion of their findings, and addressed all the concerns that I raised in my initial review. I believe that the manuscript is suitable for publication in Development.

Reviewer 3

SUMMARY OF THE ADVANCE MADE IN THIS PAPER AND ITS POTENTIAL SIGNIFICANCE TO THE FIELD

The revised manuscript had addressed most reviewer comments. New organoid studies provide potential insights that SEMFs can support ISC regeneration through inflammation signals that are TGFb dependent and cytokine/TGFb-independent processes.

SUGGESTIONS TO AUTHORS

None

© 2026. Published by The Company of Biologists under the terms of the Creative Commons Attribution License (https://creativecommons.org/licenses/by/4.0/).
